# Lectin PLL3, a Novel Monomeric Member of the Seven-Bladed β-Propeller Lectin Family

**DOI:** 10.3390/molecules24244540

**Published:** 2019-12-11

**Authors:** Lukáš Faltinek, Eva Fujdiarová, Filip Melicher, Josef Houser, Martina Kašáková, Nikolay Kondakov, Leonid Kononov, Kamil Parkan, Sébastien Vidal, Michaela Wimmerová

**Affiliations:** 1 Department of Biochemistry, Faculty of Science, Masaryk University, Kotlářská 2, 611 37 Brno, Czech Republic; faltinek@mail.muni.cz; 2 Central European Institute of Technology, Masaryk University, Kamenice 5, 625 00 Brno, Czech Republic; eva.fujdiarova@ceitec.muni.cz (E.F.); melicherfilip@mail.muni.cz (F.M.); houser@mail.muni.cz (J.H.); 3 National Centre for Biomolecular Research, Faculty of Science, Masaryk University, Kotlářská 2, 611 37 Brno, Czech Republic; 4 Department of Chemistry of Natural Compounds, University of Chemistry and Technology, Prague (UCTP), Technická 5, 166 28 Prague, Czech Republic; kasakovm@vscht.cz (M.K.); parkank@vscht.cz (K.P.); 5 N.D. Zelinsky Institute of Organic Chemistry, Russian Academy of Sciences, Leninsky Prospect 47, Moscow 119 415, Russia; nkondakov@gmail.com (N.K.); leonid.kononov@gmail.com (L.K.); 6 Institut de Chimie et Biochimie Moléculaires et Supramoléculaires, CO2-Glyco, UMR 5246, CNRS, Université Claude Bernard Lyon 1, 43 Boulevard du 11 Novembre 1918, 6922 Villeurbanne, France; sebastien.vidal@univ-lyon1.fr

**Keywords:** *Photorhabdus*, lectin, *O*-methylated saccharides, l-fucose

## Abstract

The *Photorhabdus* species is a Gram-negative bacteria of the family *Morganellaceae* that is known for its mutualistic relationship with *Heterorhabditis* nematodes and pathogenicity toward insects. This study is focused on the characterization of the recombinant lectin PLL3 with an origin in *P. laumondii* subsp. *laumondii*. PLL3 belongs to the PLL family of lectins with a seven-bladed β-propeller fold. The binding properties of PLL3 were tested by hemagglutination assay, glycan array, isothermal titration calorimetry, and surface plasmon resonance, and its structure was determined by X-ray crystallography. Obtained data revealed that PLL3 binds similar carbohydrates to those that the other PLL family members bind, with some differences in the binding properties. PLL3 exhibited the highest affinity toward l-fucose and its derivatives but was also able to interact with *O*-methylated glycans and other ligands. Unlike the other members of this family, PLL3 was discovered to be a monomer, which might correspond to a weaker avidity effect compared to homologous lectins. Based on the similarity to the related lectins and their proposed biological function, PLL3 might accompany them during the interaction of *P. laumondii* with both the nematode partner and the insect host.

## 1. Introduction

*Photorhabdus* is a genus of Gram-negative bacteria belonging to the family *Morganellaceae* [1]. These bacteria are facultative anaerobic rod-shaped microorganisms with bioluminescence ability. *Photorhabdus* is a subject of many studies due to its interesting life cycle. These bacteria have not been observed freely in the soil so far, but a monoculture of certain species of the genus *Photorhabdus* lives in the gut of nematodes of the genus *Heterorhabditis* [1,2,3]. *Photorhabdus* and *Heterorhabditis* create together a nematobacterial complex, which is highly pathogenic for a wide range of insects, especially for their larval stages [2,4]. Infective juveniles (IJs), a specialized larval stadium of the *Heterorhabditis* nematode, attack the insect larva and release the *Photorhabdus* from its intestine into the insect haemocoel. Bacteria then multiply and produce various virulence factors (e.g., adhesins, toxins, proteases, and lipases) to overcome the host immune response. The insect host is killed within 48 h due to global septicaemia [2,5,6]. In addition, *Photorhabdus* produces special nutrients that are necessary for the nematode growth [2,5,7]. *Heterorhabditis* feeds on the insect tissues as well as on the *Photorhabdus* cells and undergoes several cycles of sexual reproduction. Once the nutrients are depleted, a new generation of IJs is created, which is associated with the *Photorhabdus* and leaves the cadaver to search for its next pray [2,4,5,8].

The study of the relationship between *Photorhabdus*, nematodes, and insect can provide important information about prokaryotic involvement in two different symbioses such as mutualism and pathogenicity [2,4]. The interactions between the individual members of this complicated system are not fully understood; however, it is likely that part of them will include proteins on the cell surface. One group of such proteins are lectins, which are carbohydrate-binding proteins that have been demonstrated to play a crucial role in many physiological and pathophysiological processes [9,10]. Lectins are proteins without catalytic activity that are able to bind mono- and oligosaccharides reversibly and with high specificity [9,10,11]. Lectins are abundantly expressed in viruses, bacteria, and fungi [12]. Structural studies of lectins from opportunistic fungal and bacterial pathogens show that these lectins interact with carbohydrates on the surface of host cells and they mediate adhesion, which can lead to initiating infection [12,13,14]. It is expected that lectins may play an important role in the life cycle of the *Photorhabdus*–*Heterorhabditis* complex [15,16,17]. They can mediate mutualism interactions, be involved in pathogenicity toward insect larva, or facilitate intermediate communication within the bacterial population.

Recently, a family of structurally related lectins was discovered in the bacterial genus *Photorhabdus* [15,16,17]. These lectins share a seven-bladed β-propeller fold with multiple binding sites located between the blades. The first described member, PLL from *P. laumondii* subsp. *laumondii* (formerly known as *P. luminescens* subsp. *laumondii*), occurs as a tetramer, whereas PLL2 from the same species and PHL from *P. asymbiotica* subsp. *asymbiotica* are dimers [15,16,17]. Studies show that these carbohydrate-binding proteins can interact with insect and human immune systems. For instance, PHL binds to hemocytes [15], and PLL interacts with the nematode cuticle [16]. Moreover, PHL and PLL2 participate in the immunosuppression by the inhibition of a production of the reactive oxygen species [15,17]. These lectins exhibit affinity toward l-fucose and its derivatives; PHL can recognize also other saccharides such as d-glucose or d-galactose. Interestingly, they were both shown to interact with *O*-methylated saccharides [15,16]. *O*-methylated glycans are often present in some species of bacteria, fungi, algae, plants, and worms such as nematodes, but not in mammals nor insects [18]. Moreover, *O*-methylated glycans belong to pathogen-associated molecular patterns and represent a conserved target of the fungal and animal innate immune system [19,20].

In this article, we introduce a novel l-fucose-binding lectin, which is designated as PLL3. It is the only member of the PLL family with the seven-bladed β-propeller structure existing as a monomer. The *plu0735* gene encoding PLL3 was identified in the *P. laumondii* subsp. *laumondii* genome, and its recombinant form was produced and purified. The activity of PLL3 was assessed by hemagglutination assay and its binding abilities toward carbohydrate ligands were investigated by biophysical methods. The 3D structure of PLL3 was solved by X-ray structure analysis. The differences among homologous lectins within the PLL family are discussed.

## 2. Results

### 2.1. Production and Basic Characterization of PLL3

**Figure 1:**
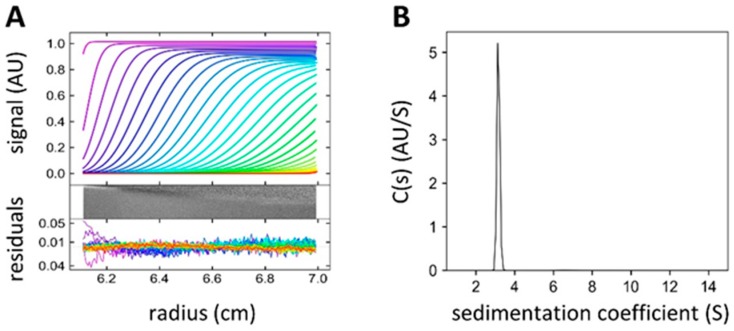
Sedimentation velocity experiment. (**A**) The sedimentation curves of PLL3 (0.23 μg mL^−1^) and fitted profiles were obtained from continuous c(s) analysis using Sedfit 15.01 [21]. (A, top) The scans were recorded every 4 min, every third scan is shown. The residual plot (A, bottom) shows the difference between experimental data and fitted curves in colored lines or in a gray bitmap. (**B**) The continuous size distribution of sedimenting species resulted in a peak with the sedimentation coefficient of 3.16 S (S_20,w_ = 3.27 S as calculated in Sednterp). The figures were created in GUSSI 1.0.8 [22].

The protein PLL3 was identified by bioinformatical analyses of the *Photorhabdus laumondii* subsp. *laumondii* genome as a close homolog of the lectins PLL [16], PLL2 [17], and PHL [15] with amino acid sequence identity from 64.5% to 66.5%. The PLL3 sequence consists of 371 amino acids (including the initial methionine) with a calculated molecular weight of 40.462 kDa. The *pll3* synthetic gene was cloned, and the protein was produced in *Escherichia coli.* The recombinant protein was purified by affinity chromatography on l-fucose-Sepharose resin and eluted isocratically with a typical yield of 40 mg per liter of medium (Figure S1A). The protein purity and identity were verified by SDS-PAGE and mass spectrometry analysis (Figure S1B–D). The oligomeric state in solution was examined using the analytical ultracentrifugation (AUC) method of sedimentation velocity. The AUC sedimentation coefficient of PLL3 was 3.27 S, which corresponds to a monomeric state of the protein (Figure 1).

### 2.2. Carbohydrate Specificity

#### 2.2.1. Hemagglutination

**Figure 2:**
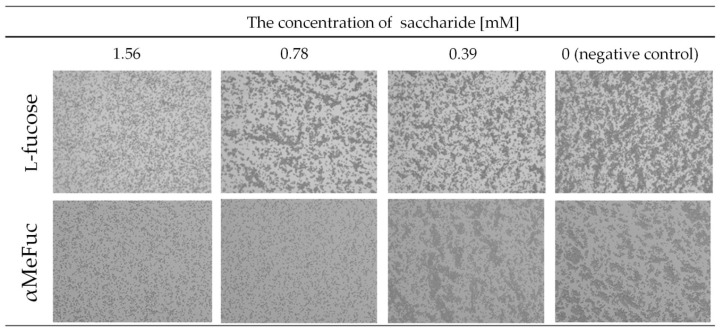
The inhibitory effect of l-fucose and methyl α-l-fucopyranoside (αMeFuc) toward the interaction of PLL3 with the erythrocytes of blood group O. The concentration of PLL3 was 50 μM in all samples; the erythrocytes were used as 10% (*v*/*v*). Carbohydrates were not added into the last sample (negative control). The lowest inhibitor concentrations at which the agglutination of erythrocytes was not still visible were 1.56 mM and 0.78 mM for l-fucose and αMeFuc, respectively. The experiment was observed at 200× magnification by optical microscope Olympus IX81.

The hemagglutination assay was used for the detection of lectin activity. PLL3 displayed very weak hemagglutination activity (50 μM) toward human erythrocytes of the blood group O, and there was no visible hemagglutination with blood groups A and B (Figure S2). The inhibition hemagglutination assay with l-fucose, methyl α-l-fucopyranoside (αMeFuc), d-galactose, d-glucose, and 3-*O*-methyl-d-glucose was also carried out. l-fucose and αMeFuc inhibited hemagglutination with a minimal inhibitory concentration (MIC) of 1.56 mM and 0.78 mM, respectively (Figure 2). The other tested saccharides did not inhibit hemagglutination at any concentration used.

#### 2.2.2. Glycan Array

**Figure 3:**
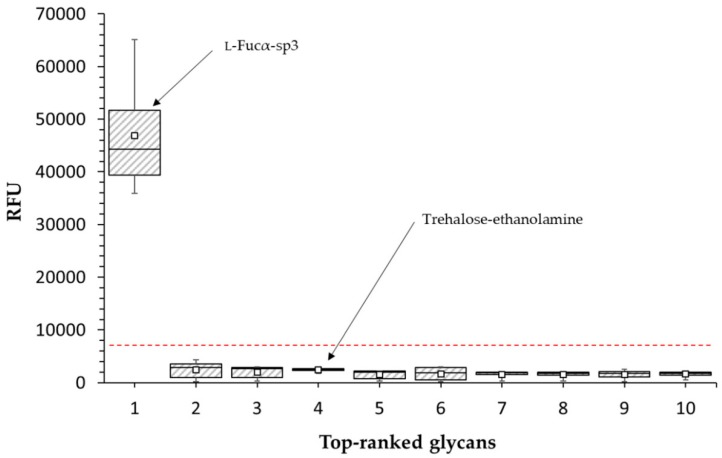
Box and whisker plot from glycan array screening with PLL3 (200 μg mL^−1^) labeled with DyLight 488 NHS Ester. The top 10 glycans including trehalose as a standard blank recommended by the manufacturer are shown. α-l-fucoside with the highest relative fluorescent response (RFU) and trehalose as the negative control are marked. The formula of linker abbreviation sp3: –O(CH_2_)_3_NH_2_. The bottom and top of the box are the first and third quartiles, the band inside the box is the median, the small square inside the box represents the mean, and the ends of the whiskers represent the minimum and maximum values of the obtained data. The red dashed line forms the boundary between the significant and non-significant response and was determined as 3 times higher than the negative control. Data from hexaplicates are shown.

A detailed screening of PLL3 binding to various glycans was performed using a glycan array microchip with more than 600 immobilized ligands consisting of different bacterial and mammalian glycans (Tables S1 and S2). The experiment revealed α-l-fucoside as the only significantly positive ligand, with a nearly 20-fold higher response than toward trehalose (a negative control recommended by the manufacturer Semiotik, Russia), while all the other tested ligands displayed a non-significant response lower than three times the response of trehalose (Figure 3).

#### 2.2.3. Isothermal Titration Calorimetry (ITC)

**Figure 4:**
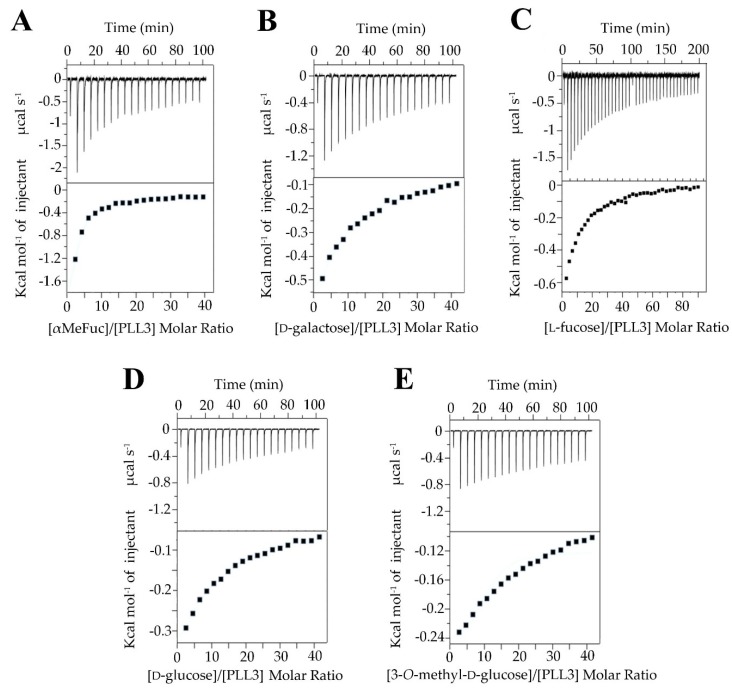
Isothermal titration calorimetry of PLL3 with methyl α-l-fucopyranoside (**A**), d-galactose (**B**), l-fucose (**C**), d-glucose (**D**), and 3-*O*-methyl-d-glucose (**E**). For each experiment, the concentration of PLL3 was 0.1 mM, and the concentration of each monosaccharide was 20 mM. Twenty injections (40 injections in the case of l-fucose) of 2.0 μL of saccharide solution were added every 300 s into the cell containing PLL3. Bottom plots show the total heat released as a function of total ligand concentration for the titration shown in the upper plots.

The thermodynamic parameters of PLL3 binding toward selected carbohydrates were evaluated by isothermal titration calorimetry (Figure 4). The experiment was carried out with five monosaccharides: l-fucose, methyl α-l-fucopyranoside, d-galactose, d-glucose, and 3-*O*-methyl-d-glucose. Due to the low-affinity interaction, the stoichiometry was fixed based on homologous PHL and PLL binding site occupancies to integer values ranging from 3 to 7, and the corresponding K_D_ value was calculated (Table 1). αMeFuc was discovered as the best binding partner with K_D_ in the submillimolar range. Four to five times higher equilibrium dissociation constants were determined for d-galactose, l-fucose, and d-glucose. 3-*O*-methyl-d-glucose appeared to be the weakest binding partner with K_D_ values above 10.4 mM.

**Figure 5:**
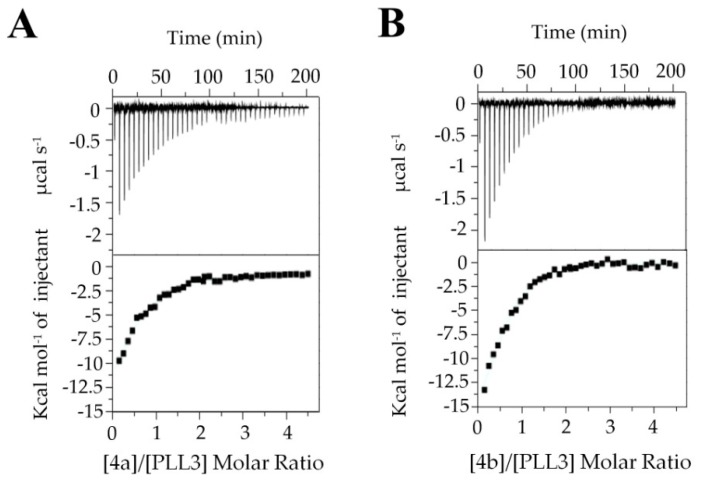
Isothermal titration calorimetry of PLL3 with decavalent fucosylated glycoclusters **4a** (**A**) and **4b** (**B**). For each experiment, the concentration of PLL3 was 0.1 mM, and the concentration of both fucosides was 1 mM. Forty injections of 2.0 μL of ligand solution were added every 300 s into the cell containing PLL3. Bottom plots show the total heat released as a function of total ligand concentration for the titration shown in the upper plots.

To investigate the probable oligovalent nature of PLL3/saccharide interaction, the ITC experiments with synthetic decavalent fucosylated glycoclusters were performed. Two pillar[5]arene-based glycocompounds, which were designated **4a** and **4b**, were used (Figure 5). Both multivalent ligands exhibited higher affinity than in the case of individual monosaccharides. **4b** fucoside with shorter linkers was a slightly better binder than **4a**. The equilibrium dissociation constants were 57 μM and 36 μM with stoichiometry values of approximately 0.5 and 0.6 for **4a** and **4b**, respectively.

#### 2.2.4. Surface Plasmon Resonance (SPR)

The surface plasmon resonance experiments were carried out to investigate PLL3 interaction with immobilized glycans. These experiments revealed that PLL3 interacts both with immobilized α-l-fucoside and *O*-methylated disaccharide 3,6-*O*-Me_2_-Glcβ1-4(2,3-*O*-Me_2_)Rhaα (OMeDis). The PLL3 interacting with α-l-fucoside and OMeDis gave an apparent K_D_ value of 322 ± 86 μM and 607 ± 46 μM, respectively. l-fucose proved to be a stronger binding partner even though the direct immobilization of OMeDis with the higher surface density allowed for a stronger avidity effect, while streptavidin–biotin immobilization of l-fucose favors one-to-one binding. Different ways of immobilization and hence the surface density of these ligands resulted also in a difference in the maximal theoretical response for each channel. Due to a higher relative response and weaker affinity, the OMeDis channel was chosen for competitive inhibition tests. The interaction between the lectin and immobilized OMeDis was affected by the addition of monosaccharides, allowing for determination of the inhibition constants IC_50_ (Table 2). The best inhibitor was shown to be αMeFuc followed by the native l-fucose with IC_50_ values in the low millimolar range. 3-*O*-methyl-d-glucose was a weak inhibitor with IC_50_ being 10 times higher in comparison with l-fucose. On the contrary, d-glucose did not exhibit any inhibitory effect, even at the highest concentration used.

### 2.3. X-Ray Structure of PLL3

**Figure 6:**
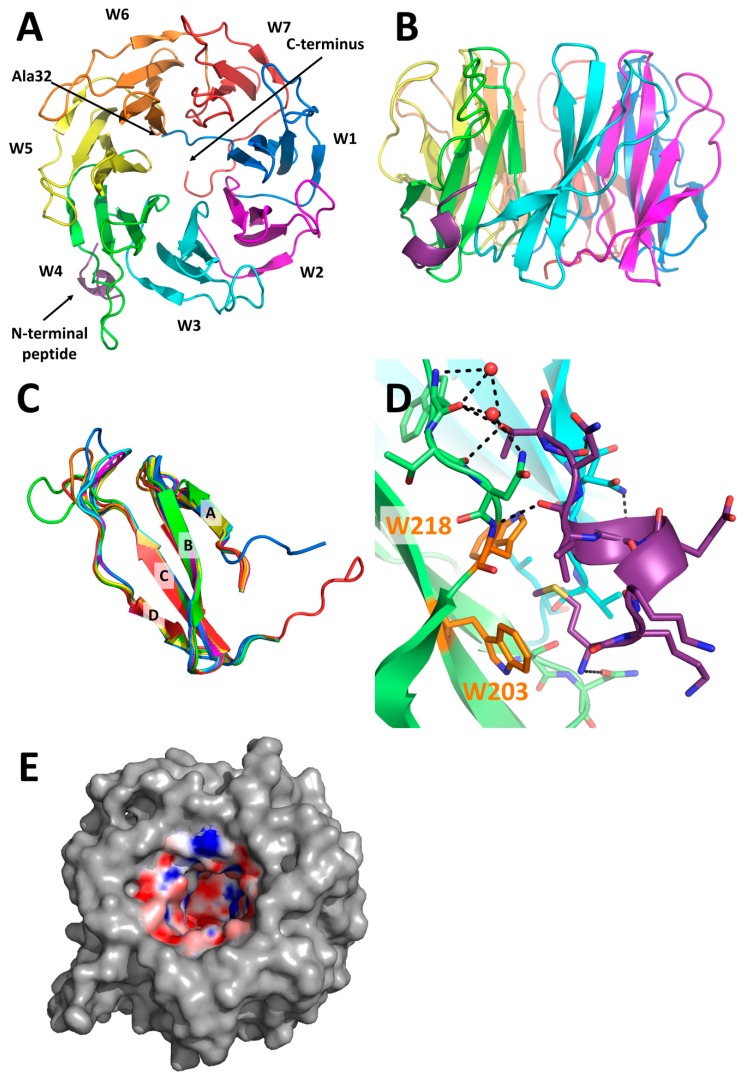
Structural features of PLL3. (**A**) Seven-bladed β-propeller fold of monomeric PLL3 (top view). Repeating W-motifs are labeled and represented by a different color. A short N-terminal peptide is represented in purple. (**B**) Side view of the monomer. (**C**) Structural alignment of all seven W-motifs of the PLL3 lectin. Color assignment is in the same manner as in Figure 6A. (**D**) The N-terminal peptide (in purple) interacts with the monomer in a groove between blades W3 (cyan) and W4 (green). Water molecules involved in the interaction are shown as red spheres, and polar contacts are represented as black dashes. Trp residues interacting with Met1 via CH–π interaction are highlighted in orange. (**E**) Charge distribution inside the cavity of the PLL3 monomer (negative potential in red, positive potential in blue, and neutral potential in white).

The 3D structure of PLL3 was solved by X-ray crystallography using the molecular replacement method. The protein crystallized as a monomer in the *P*212121 space group with one monomer per asymmetric unit, and it exhibits a seven-bladed β-propeller fold (Figure 6A,B). Statistics from the data collection and structure refinement are listed in Table 3. The monomer has a continuous well-defined electron density from Ala32 to the C-terminal amino acid Lys371. N-terminal and C-terminal parts of the monomer interact with loops connecting blades, and they are found in a close proximity in the crystal structure. Overall, the shape of the molecule is a torus with a height of 29 Å and a diameter of 47 Å. Blades, also called W-motifs, are arranged around the seven-folded pseudo-axis, and they consist of four twisted antiparallel β-strands (A-B-C-D) connected by loops (Figure 6C). The structural alignment of all W-motifs revealed their high similarity with root mean square deviation (RMSD) 0.21–0.70 Å. The most evident difference amongst all blades is in the loop between the C and D strands of the W4 motif, which has a different orientation than the other aligned loops.

Electron density corresponding to the first eight amino acids was found in the hydrophobic pocket between the W3 and W4 motifs. The side chain of the initial Met1 is stabilized by interaction with Trp203 and Trp218. This N-terminal peptide forms a short α-helix that interacts via hydrogen bonds and water bridges with a groove between motifs W3 and W4 (Figure 6D), which may contribute to a conformational change of the loop between the C and D strands of motif W4.

An inner cavity is formed inside the torus by the A strands of every W motif. The bottom of the cavity is established by the C-terminal and N-terminal parts of the monomer. The diameter of the cavity ranges from 18 Å to 12 Å. The nature of the cavity is mostly hydrophilic, but it also contains hydrophobic patches (Figure 6E). No other electron density than that corresponding to water molecules was found inside this cavity.

**Figure 7:**
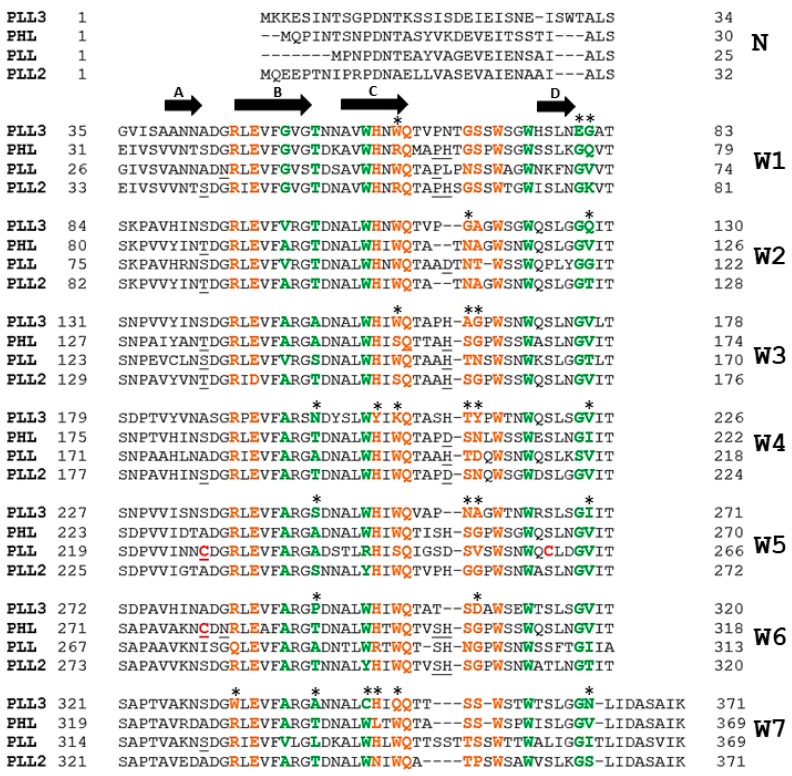
Sequence alignment of PLL3 (NCBI reference number: WP_011145110.1) with PHL (NCBI reference number: WP_012776886.1), PLL (NCBI reference number: WP_011145107.1), and PLL2 (NCBI reference number: WP_011145109.1). The residues corresponding to the polar binding sites are highlighted in orange, and the residues corresponding to the hydrophobic binding sites are highlighted in green. Cysteine residues involved in oligomerization of the PHL and PLL lectins are highlighted in red. Residues involved in the dimerization interface of PHL, PLL, and PLL2 are underlined. Binding site residues differing between PLL3 and PHL are marked with an asterisk. Full arrows represent positions of the β-strands (A-B-C-D). N-terminal parts of the proteins (N) and individual W-motifs (W1–W7) are labeled.

The PLL3 structure is highly similar to homologous lectins from the PLL family, with the structural alignment RMSD value for monomers being 0.743 Å (PHL, PDB: 5MXH), 0.647 Å (PLL, PDB: 5C9P), or 0.596 Å (PLL2, PDB: 6RG2). The main differences are in the positions of loops in between individual blades. The co-crystallization and soaking experiments with sugars did not result in the structure of the PLL3/ligand complexes. Within the PLL family, the presence of up to seven polar and seven hydrophobic binding sites per monomer was considered, with the binding sites located in between adjacent blades. The sequence and structural comparison of the putative binding sites of PLL3 and the verified glycan-binding sites of its homologs revealed a probable retaining of five polar and four hydrophobic binding sites in PLL3 (Figure 7, Figure S3 and S4). Regarding the polar binding sites of PLL3, the putative sites 1P and 2P are fully conserved to the PHL site 2P, while binding sites 3P, 5P, and 6P have a different orientation of the loop, which is involved in an interaction with O4 oxygen of d-galactose in PHL. Significant differences in the amino acid composition were observed in the putative sites 4P and 7P. These binding sites do not contain a tryptophan residue, which is involved in the CH–π stacking interaction and is important for stabilization of the saccharide molecule. 

The putative hydrophobic binding sites 2H, 3H, 4H, and 7H of PLL3 show only a small variability in amino acid composition compared to the binding site 6H of PHL. Other potential PLL3 hydrophobic binding sites have more severe amino acid changes, such as loss of the tryptophan residue involved in the CH–π stacking interaction (binding site 6H) or hindrance of the binding site by glutamic acid (binding site 1H). In putative site 5H, the key interacting residues are present; however, the replacement of Thr for hydrophobic Pro293 may affect the ligand binding. In the PLL3 crystal structure, the putative binding site 3H is occupied by a short N-terminal peptide with Met1 being coordinated in the binding pocket.

Using the multiple sequence alignment, the analysis of the key residues involved in the dimerization interaction at homologous lectins PHL, PLL, and PLL2 was performed (Figure 7). The main difference to the PHL and PLL interfaces is a lack of Cys residue in position 235 (PLL3) or 280 (PLL3), respectively, disabling the disulfide bond formation. In comparison to PLL2, two Ser residues are mutated to Ala in PLL3 (positions 43 and 187) and the loop C–D of blade W6 is shorter due to lacking His residue. Together with other differences in the C–D loops, this can explain the monomeric state of PLL3.

## 3. Discussion

We have characterized protein PLL3, coded by gene *plu0735*, from bacterium *Photorhabdus laumondii* subsp. *laumondii*, which is a highly virulent insect pathogen. The protein was discovered as a novel member of the PLL family with a seven-bladed β-propeller fold. In contrast to already described lectins, PLL3 exists as a monomer with a molecular weight of 40.4 kDa. 

PLL3 was proved to be an l-fucose-binding lectin, similarly to its close homologs from the family [15,16,17]. In solution, PLL3 also exhibits millimolar affinity toward other monosaccharides (e.g., αMeFuc, d-glucose, d-galactose). Weak affinity is generally observed for lectin monosaccharide interactions [10]. In addition, the preference for αMeFuc over l-fucose was frequently observed for other lectins including PLL, PLL2, and PHL [14,15,16,17]. Even though the experiments in solution revealed potential lectin promiscuity with respect to a recognized ligand, the screening of more than 600 glycans using glycan array microchips showed significant PLL3 binding only to the α-l-fucoside. A discrepancy between the interaction of free and surface-bound molecules is common [23,24] and was described previously also within the PLL family (e.g., PHL binding of d-galactose [15]). On the other hand, a strict preference for a simple monosaccharide (l-fucose) is quite unusual, especially since the fucose unit is present in dozens of other tested epitopes. This can mean that the presence of branched epitopes markedly decreases the binding of PLL3. That would correspond well to the fact that PLL3 can agglutinate only the blood group O erythrocytes with the straight terminal antigen H and not the other blood groups with branched terminal glycans. Nevertheless, the natural binding partner of PLL3 can be a fucosylated oligosaccharide of insect or nematode origin that was not present in the tested set. Fucosylated oligosaccharides were identified in both insects [25,26] and nematodes [27]; however, the glycobiology of these organisms is not well investigated yet.

A weak but unambiguous binding toward 3-*O*-methyl-d-glucose and the unusual *Mycobacterium leprae* disaccharide 3,6-*O*-Me_2_-d-Glcβ1-4(2,3-*O*-Me_2_)-l-Rhaα (OMeDis) [28,29] was observed. *O*-methylated glycans are often present in some species of bacteria, fungi, algae, plants, and nematodes, but they were not identified in mammals and insects. Moreover, they constitute a conserved target of the fungal and animal innate immune system [18,19]. The interaction between PLL3 and immobilized OMeDis was 10^5^-fold weaker than for PLL2 and PHL [17], demonstrating that this particular saccharide is not likely to be a natural binding partner of PLL3. However, the general ability of PLL3 to recognize *O*-methylated saccharides is worth noting.

Unlike other described seven-bladed β-propeller lectins from *Photorhabdus* bacteria, PLL3 is a monomer in solution as well as in the crystal structure. The oligomerization of lectins PHL and PLL is stabilized by the formation of disulfide bridges between monomers [15,16]. PLL3 does not contain Cys in the corresponding positions (Figure 7). The association of the two monomers of PLL2 is caused mainly by hydrogen bonds [17]. The absence of the key residues at the dimerization interface (Figure 7) may lead to the loss of the PLL3 ability to form oligomers.

The N-terminus of all known members of the PLL-family is highly flexible. However, the crystal structure of the PLL3 lectin revealed a short N-terminal peptide with the initial methionine located in the potential hydrophobic binding site. The orientation of the N-terminal peptide and the location of the first resolved amino acid of the interacting β-propeller suggest that they both belong to two nearby monomers in the crystal structure. Hence, the N-terminal peptide might contribute to the crystal packing and at the same time interfere with carbohydrate binding. This may explain the inability of PLL3 crystals to be formed in the presence of a ligand. Until now, there has only been one reported structure within the PLL family with a resolved N-terminus—the lectin PHL from *P. asymbiotica* with a short N-terminal tripeptide located in the putative fucose binding site between motifs W7 and W1 [15]. This shows that the binding specificity of such lectin hydrophobic binding sites can be relatively broad and may not be restricted to carbohydrate-like ligands.

As was previously reported, PHL possesses two types of binding sites with different binding specificities [15]. The sequential and structural comparison of the PHL binding sites and the putative binding sites of PLL3 revealed a probable retaining of five polar sites and four hydrophobic sites. In general, the amino acids of PLL3 are highly conserved and similar to other binding sites within the lectin family. However, several differences in single residues are likely to affect the ligand binding. The existence of multiple binding sites within PLL3 was supported by binding experiments. ITC measurements revealed increased affinity toward the multivalent fucosylated compounds compared to l-fucose, which can be explained by the presence of a weak avidity effect. In addition, K_D_ for immobilized l-fucose was lower than for the free monosaccharide. This corresponds to the existence of several fucose-binding sites per the PLL3 molecule. Based on homology to PHL and the SPR inhibition tests, we suspect the hydrophobic sites to recognize both l-fucose and *O*-methylated saccharides. 

The functional and structural characterization of PLL3 showed this lectin as a member of the PLL family. The binding affinity of PLL3 toward studied saccharides, e.g., l-fucose and *O*-methylated saccharides, is lower compared to homologous lectins. However, the specificity toward surface saccharides is very narrow. Together with the monomeric nature of PLL3, this could mean that its role in the *Photorhabdus* life cycle is more targeted than that of other described PLL3 homologs. The homologs interfere with the host innate immune system [15,17], and we can assume the PLL3 might play a similar role in the bacterial life cycle as well. The high complexity of lectins identified in genus *Photorhabdus* would be an interesting subject for a broad comparative study. A better understanding of lectins role in the *Photorhabdus*–*Heterorhabditis* interactions may be also used to improve the further application of this biological insecticide.

## 4. Material and Methods

### 4.1. Materials

l-fucose was purchased from AppliChem (Darmstadt, Germany). Methyl α-l-fucopyranoside, d-glucose, and d-galactose were purchased from Carbosynth (Compton, UK). 3-*O*-methyl-d-glucose, biotin, and streptavidin were purchased from Sigma-Aldrich (St. Louis, MO, USA). Biotinylated α-l-fucoside was purchased from Synthaur LLC (Moscow, Russia). Methylated disaccharide 3,6-*O*-Me_2_-d-Glcβ1-4(2,3-*O*-Me_2_)-l-Rhaα-*O*-(*p*-C_6_H_4_)-*O*-CH_2_CH_2_NH_2_ was synthesized as described previously [29]. DyLight 488 NHS ester was purchased from ThermoScientific (Rockford, MI, USA). Protein molecular Marker III was purchased from AppliChem (Darmstadt, Germany). Other chemicals were purchased from Sigma-Aldrich, Duchefa, ForMedium and Applichem companies.

### 4.2. PLL3 Identification and Cloning

Bioinformatic analyses of the genome of *Photorhabdus laumondii* subsp*. laumondii* TT01 (Taxonomy ID: 243265, NCBI reference sequence: BX470251.1) [6] was performed using the NCBI BLAST tool [30]. The sequence of the PLL lectin from *Photorhabdus laumondii* (Uniprot ID: Q7N8J0) was used as a template for the analyses. The hypothetical protein Plu0735 (Uniprot ID: Q7N8I7) was identified as a PLL homolog and was designated PLL3. A synthetic gene named *pll3* was prepared by Life Technologies (Thermo Fisher Scientific, Waltham, MA, USA) with the codon optimized for expression in *E. coli*. HindIII and NdeI restriction endonuclease sites were added to the sides of the sequence. The gene was introduced into the pET25b (Novagen, Germany) vector using HindIII and NdeI restriction enzymes (NEB). The created vector pET25b_*pll3* was multiplicated in *E. coli* DH5α and subsequently cloned into the expression strain *E. coli* Tuner (DE3) (Novagen, Germany). The presence of the pET25b_*pll3* vector in transformed cells was ensured by the introduced ampicillin resistance. The sequence of pET25b_*pll3* was confirmed by sequencing the re-isolated vector.

### 4.3. PLL3 Purification

Cells were disrupted by ultrasonic disintegration (VCX 500, Sonics & Materials, Inc., Newton, CT, USA). The cell lysate was separated by centrifugation at 21,000 g at 4 °C for 1 h and filtrated through a filter with pore size 0.22 µm (CarlRoth, Karlsruhe, Germany). Then, the cell lysate was loaded onto l-fucose-Sepharose resin equilibrated with 20 mM Tris/HCl, pH 7.5 buffer. The PLL3 protein was purified by affinity chromatography using the ÄKTA FPLC system (GE Healthcare, Buckhinghamshire, UK) and eluted isocratically. The purity of eluted fractions was analyzed using SDS-PAGE electrophoresis (12% gel stained with Coomassie Brilliant Blue R-250 or with silver nitrate). The purified protein was dialyzed against the buffer suitable for further studies.

### 4.4. Analytical Ultracentrifugation (AUC)

The oligomeric state of PLL3 in solution was investigated by AUC using a ProteomeLab XL-A analytical centrifuge (Beckman Coulter, Brea, CA, USA) equipped with an An-60 Ti rotor. Prior to analyses, the protein was dialyzed against the working buffer (20 mM Tris/HCl, 150 mM NaCl, pH 7.5), and the dialysate was used as an optical reference. Experiments were performed at different protein concentrations (0.03–0.23 mg mL^−1^). Sedimentation velocity experiments were conducted in titanium double-sector centerpiece cells (Nanolytics Instruments, Germany) loaded with 380 µL of the protein and 380 μL of the reference solution. Data were collected at 20 °C at a rotor speed of 50,000 rpm. Scans were performed using absorbance optics at 4 min intervals at 280 nm and 0.003 cm spatial resolution in a continuous scan mode. The partial specific volume of the protein together with solvent density and viscosity were calculated from the amino acid sequence and the buffer composition, respectively, using the software Sednterp (http://jphilo.mailway.com/index.htm). The sedimentation profiles were analyzed with the program Sedfit 15.01 [21]. A continuous size distribution model for non-interacting discrete species was used to provide a distribution of sedimentation coefficients.

### 4.5. Hemagglutination

Experiments of hemagglutination and its inhibition were performed using anonymized human erythrocytes of blood groups A, B, and O (obtained from the Transfusion and Tissue Department, The University Hospital Brno, Brno, Czech Republic) stabilized by 3.8% sodium citrate. The erythrocytes were washed three times by phosphate-buffered saline (PBS) (137 mM NaCl, 2.7 mM KCl, 8 mM Na_2_HPO_4_, 1.5 mM KH_2_PO_4_, pH 7.4) and diluted to 50% solution by the same buffer. Erythrocytes were stabilized by 0.01% (*w*/*v*) NaN_3_ and stored at 4 °C until further use. Prior to the experiment, erythrocytes were treated by 0.1% papain for 30 min at room temperature and subsequently washed three times by PBS buffer. Hemagglutination experiments were performed according to the procedure described previously [31]. Erythrocytes were mixed with serially diluted PLL3 solutions in a ratio of 1:1 (*v*/*v*) to the final concentration of erythrocytes of 10% (*v*/*v*) and to the final concentrations of the PLL3 ranging between 50 and 0.05 μM. The mixture was incubated for 30 min at room temperature, and each sample was observed under the optical microscope (Olympus IX81 with camera DP72; 200x magnification; SW CellSens Dimension, Olympus corporation, Tokyo, Japan).

For the hemagglutination inhibition assay, the PLL3 was mixed with serially diluted saccharides (l-fucose, αMeFuc, d-galactose, d-glucose, and 3-*O*-methyl-d-glucose) dissolved in PBS buffer, and mixtures were incubated for 15 min at room temperature. Subsequently, erythrocytes of blood group O were added into the mixture with following incubation for 15 min. The final concentration was 50 μM for the PLL3 and 10% (*v*/*v*) for erythrocytes. Concentrations of l-fucose and αMeFuc ranged from 50 to 0.05 mM. Concentrations of d-galactose, d-glucose, and 3-*O*-methyl-d-glucose ranged from 100 to 0.1 mM. Each sample was observed under the optical microscope (Olympus IX81 with camera DP72; 200x magnification; SW CellSens Dimension, Olympus corporation, Tokyo, Japan).

### 4.6. Glycan Array

Purified PLL3 protein was labeled with DyLight 488 NHS Ester (Thermo Scientific, Rockford, MI, USA) according to the manual instructions of the manufacturer. The unbound dye was removed from the solution using the Zeba Spin Desalting Column (Thermo scientific). The labeled lectin was used for glycan array screening using a glycan microarray chip (Semiotik, Moscow, Russia; chip format OSPS280715; slide number 10085636) containing over 600 mammalian and bacterial glycans (all in hexaplicates). The glycan microarray chip was incubated in 1.0 mL of PBS buffer supplemented with 0.1% (*v*/*v*) Tween 20 and placed in a humid chamber for 15 min (37 °C, 40 rpm). Then, the buffer was replaced by 0.5 mL PLL3 (200 μg mL^−1^) diluted in PBS buffer with 0.1% Tween 20 and 1% (*w*/*v*) bovine serum albumin (Serva, Germany). After incubation (90 min, 37 °C, 40 rpm), the chip was washed by decreasing concentrations of PBS with 0.1% Tween 20, followed by multiple immersions in ultrapure water. Subsequently, the chip was dried by air blow. Fluorescence was read with a scanner InnoScan 1100 AL (Innopsys, Carbonne, France) with a 488 nm laser at 20 °C. The data were analyzed with the software Mapix 8.2.2 and an online glycan chip converter (Semiotik, https://rakitko.shinyapps.io/semiotik). The relative fluorescent response of PLL3 binding was calculated from hexaplicates of each glycan immobilized onto the chip. Only glycans with a response three or more times higher than trehalose and concurrently with a signal-to-noise ratio higher than two were considered as significant binders.

### 4.7. Isothermal Titration Calorimetry

ITC experiments were carried out using an AutoITC200 calorimeter (Malvern, UK) at 25 °C. The purified lectin was dialyzed against 20 mM Tris/HCl, 150 mM NaCl, pH 7.5 buffer before measurements. Individual ligands (l-fucose, αMeFuc, d-galactose, d-glucose, 3-*O*-methyl-d-glucose, and decavalent fucosylated glycoclusters **4a** and **4b**) were dissolved in the same buffer. PLL3 (100 μM) in the cell was titrated by the successive addition (2.0 μL) of the ligand in the syringe (20 mM for monosaccharides, 1 mM for multivalent fucosides). Experiments with monosaccharides were performed at stirring 750 rpm with a reference power of 10 μcal s^−1^. Experiments with multivalent fucosides were performed at stirring 500 rpm with a reference power of 6 μcal s^−1^. Titrations were carried out in three independent measurements for each ligand. Blank measurements were subtracted from collected data. For multivalent fucosides, two blank measurements were performed: titration of the buffer into the cell with PLL3 and titration of the buffer into the buffer. For monosaccharides, the additional blank measurement, the titration of a given ligand into the cell with buffer, was performed. The data were evaluated in software MicroCal PEAQ-ITC Analysis Software (Malvern, UK) by using the global fit method. Integrated heat effects were analyzed by nonlinear regression.

### 4.8. Surface Plasmon Resonance

SPR experiments were performed in a BIAcore T200 instrument (GE Healthcare, UK) using a four-channel sensor chip CM5 with a carboxymethyldextran surface layer (GE Healthcare, UK). Saccharides were immobilized on the chip surface using the procedure as described elsewhere [17]. Immobilization was performed in the HBS buffer (10 mM HEPES, 150 mM NaCl, 0.05% Tween 20, pH 7.5) at a flow rate of 10 μL min^−1^. For OMeDis immobilization, the surface of the channel was activated with *N*-hydroxysuccinimide/*N*-ethyl-*N*-(3-dimethylaminopropyl)carbodiimide solution (NHS/EDC) and subsequently OMeDis covalently modified by linker (-*O*-(*p*-C_6_H_4_)-OCH_2_CH_2_NH_2_) in HBS was injected. The final response of immobilized OMeDis was 136 RU. Non-reacted groups were blocked by 1 M ethanolamine/HCl (pH 8.5). A corresponding blank channel was prepared the same way, omitting the ligand injection. For α-l-fucoside immobilization, the NHS/EDC activated surface was modified by streptavidin to a final response of 3150 RU and subsequently blocked by 1 M ethanolamine HCl (pH 8.5). Biotinylated α-l-fucoside was injected onto this channel to the final response of 63 RU. A corresponding blank channel was prepared the same way without the injection of biotinylated fucoside.

The determination of apparent K_D_ was performed simultaneously in all channels using 20 mM Tris/HCl, 150 mM NaCl, 0.05% Tween 20, and pH 7.5 running buffer. Between individual injections, the channels were regenerated by 50 mM NaOH for 30 s. The experiment was run at a flow rate of 30 μL min^−1^ and a contact time of 120 s. The concentration of PLL3 ranged from 100 μM to 0.195 μM. An apparent K_D_ was calculated in BIAevaluation software using the steady-state approach.

SPR inhibition tests were carried out on a channel with immobilized OMeDis using the same buffer and flow rate values as those for the binding studies. The final concentration of the lectin was 20 μM. PLL3 was mixed with serially diluted saccharides (50–0.05 mM for l-fucose; 12.5–0.013 mM for αMeFuc; 200–0.2 mM for 3-*O*-methyl-d-glucose; and 400–0.4 mM for d-glucose and d-galactose) and injected onto the chip. The lectin sample without ligand was used as a control with 0% inhibition effect. The response of blank channel as well as the response of corresponding saccharides were subtracted from the response of lectin bound to the OMeDis surface at equilibrium. A final response was plotted against the concentration of inhibitor in order to determine IC_50_ (concentration of inhibitor resulting in 50% inhibition of binding).

### 4.9. Crystallization

Purified PLL3 was concentrated to 14.4 mg mL^−1^ using an ultrafiltration unit (Milipore) with 10 kDa cut-off filters (Milipore). The commercial screening kits PACT, Classic Lite, Classic, Classic II (Qiagen, Hilden, Germany) were used to find initial crystallization conditions utilizing the sitting drop vapor diffusion method at 20 °C. The initial hits were further optimized in 96-well format (96 well 3-Drop Swissci Plate, Molecular Dimention, Sheffield, UK). The best diffracting crystals were obtained under following conditions: 200 nL drops with 2:1, 1:1, and 1:2 ratios of protein/precipitant (0.1 M HEPES, pH 7.5, 10% PEG 8000). Crystals were soaked in 40% PEG 400 as a cryoprotectant and frozen in liquid nitrogen. The attempts to crystallize PLL3 in the presence of the binding partner were performed by adding saccharide (100 mM final concentration) to PLL3 at final concentration 12.95 mg mL^−1^ prior to mixing with precipitant.

### 4.10. Data Collection and Structure Determination

Diffraction data of PLL3 were collected on beamline MX 14.1 at the BESSY II electron storage ring (Berlin, Germany). Collected images were processed by XDSAPP [32] and converted to structural factors using the program package CCP4 v.7.0 [33]. Structural factors were scaled with 5% of data reserved for R_free_ factor calculation. The phase problem was solved by the molecular replacement method using MOLREP [34] with monomeric coordinates of the PLL lectin (PDB: 5C9P) used as an initial model. Real space refinement of the model was carried out by COOT [35] and reciprocal space refinement was performed by REFMAC5 [36]. Water molecules were placed using Coot and inspected manually. The final model was validated in a PDBe validation server (http://pdbe.org) and deposited with PDB ID 6T96.

### 4.11. Synthesis of Decavalent Fucosides

**Figure 8:**
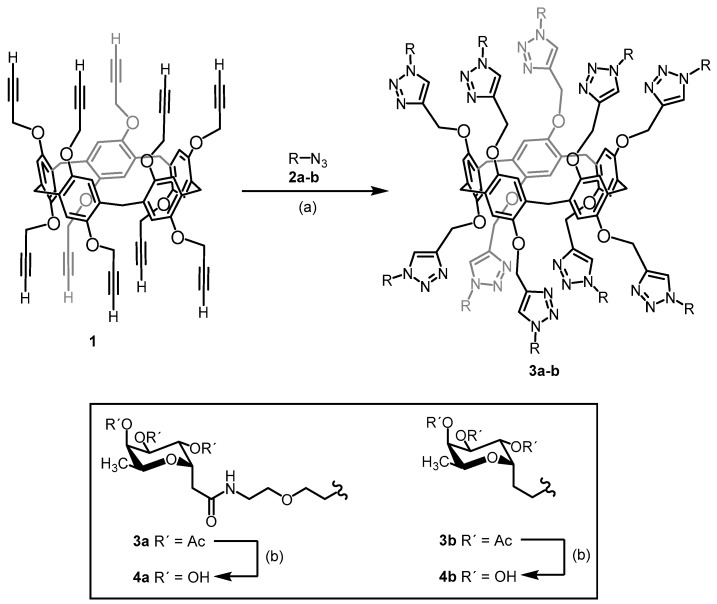
Synthesis of pillar[5]arene-based glycoclusters **4a** and **4b**. (a) Conjugation of (propargyl)_10_ pillar[5]arene-based glycoclusters (**1**) with azide **2a** or **2b** in the presence of CuSO_4_•5 H_2_O, sodium ascorbate, and CH_2_Cl_2_/H_2_O (1:1) resulting in the synthesis of compounds **3a** and **3b**, respectively. (b) Deprotection of the acetyl groups from compounds **3a** and **3b** in the presence of Et_3_N and MeOH/H_2_O (4:1), resulting in the synthesis of the desired glycoclusters **4a** and **4b**, respectively. Achieved yields: 70% (**3a**), 85% (**3b**), 98% (**4a**) and 94% (**4b**).

The synthesis of the pillar[5]arene-based glycoclusters was based on the Cu(I)-catalyzed azide–alkyne cycloaddition (CuAAC) [37,38,39,40,41,42,43,44,45,46,47,48] of propargyl-functionalized pillar[5]arene-based glycoclusters with two azido-functionalized *C*-l-fucosides with different linkers. The procedure is described in detail in the Supporting Information section (File S1). In brief, the conjugation of *N*-(5-azido-3-oxa-pentyl)-2-(2,3,4-tri-*O*-acetyl-α-l-fucopyranosyl)ethanamide (**2a**) or 2-(2,3,4-tri-*O*-acetyl-α-l-fucopyranosyl)ethylazide (**2b**) [39] with (propargyl)_10_ pillar[5]arene-based glycoclusters (**1**) [46] provided the acetylated glycoclusters **3a** and **3b,** respectively (Figure 8). The compounds were synthesized in 70–85% yield as a 1:1 mixture of diastereomers. For these macromolecules, the separation was unsuccessful even after several flash column chromatographies on silica gel. Although these isomeric mixtures could affect the interpretation of the binding studies toward lectins, the spatial arrangements of the carbohydrate epitopes for decavalent scaffolds will not differ greatly, and the general presentation of the binding partners to lectins will be statistically homogenous. Finally, deprotection of the acetyl-protecting groups afforded the desired water-soluble glycoclusters **4a** and **4b** in nearly quantitative yields of 94–98%. All the new compounds were fully characterized by ^1^H and ^13^C-NMR spectroscopy (File S1). Their structures were further confirmed by mass spectrometry.

## 5. Conclusions

PLL3 was identified in *Photorhabdus laumondii* subsp. *laumondii* as a novel member of the PLL lectin family. Unlike its homologs, it exists in a monomeric form and displays a weak but unusually narrow specificity toward immobilized saccharides. While its natural ligand is not known, it is likely to be a fucose-based oligosaccharide or yet unrevealed complex molecule, such as a saccharide with *O*-methylation. Therefore, the biological role of PLL3 in the *Photorhabdus* life cycle might be quite specific, and its study may be important for deciphering the complicated relations in the *Photorhabdus*–*Heterorhabditis*–insect symbiosis.

## Figures and Tables

**Table 1:** The calculated K_D_ values (in mM) for the interaction between PLL3 and individual monosaccharides determined by isothermal titration calorimetry (ITC). Standard deviations were calculated from three independent measurements.

Ligand	Stoichiometry				
	3	4	5	6	7
methyl α-l-fucopyranoside	0.7 ± 0.02	0.7 ± 0.03	0.6 ± 0.03	0.6 ± 0.03	0.6 ± 0.03
d-galactose	2.8 ± 0.14	2.7 ± 0.14	2.6 ± 0.13	2.5 ± 0.13	2.3 ± 0.13
l-fucose	3.4 ± 0.12	3.3 ± 0.12	3.2 ± 0.11	3.1 ± 0.10	3.0 ± 0.09
d-glucose	3.7 ± 0.08	3.6 ± 0.08	3.4 ± 0.08	3.3 ± 0.07	3.1 ± 0.07
3-*O*-methyl-d-glucose	11.3 ± 1.55	11.1 ± 1.50	10.9 ± 1.50	10.7 ± 1.50	10.4 ± 1.45

**Table 2:** Inhibitory effect of individual monosaccharides on the interaction between PLL3 and immobilized OMeDis. IC_50_ was computed from a plot of serial dilutions vs. % of inhibition.

Ligand	IC_50_ [mM]
methyl α-l-fucopyranoside	1.04 ± 0.05
l-fucose	2.51 ± 0.09
3-*O*-methyl-d-glucose	26.36 ± 2.84
d-galactose	156.46 ± 53.87
d-glucose	>400

**Table 3:** Statistics from data collection and refinement for PLL3. Values in parentheses correspond to the highestresolution shell.

**Data Collection**	
Protein Data Bank Code	6T96
Space group	*P*212121
Wavelength (Å)	0.91842
Unit cell parameters (Å)	
a/b/c	56.58/69.42/76.71
α/β/γ	90/90/90
Resolution range (Å)	51.46–1.65 (1.74–1.65)
Total number of observations	271406
Unique reflections	37089
Completeness (%)	99.9 (99.2)
R_merge_	0.143 (0.989)
CC_1/2_ (%)	99.7 (70.8)
Multiplicity	7.3 (7.4)
<*I*/σ(*I*)>	8.2 (1.7)
**Refinement Statistics**	
Refine resolution (Å)	45.53–1.65
Reflection used	35215
Reflection used for R_free_	1804
R_work_ factor (%)	16.93
R_free_ factor (%)	19.60
Root mean square deviation (RMSD)	
Bond lengths (Å)	0.009
Bond angles (degree)	1.48
Chiral volumes (Å^3^)	0.073
Number of non-hydrogen atoms (total)	3114
Number of water molecules	325
Ramachandran plot (%)	
Residues in most favorable regions	96.6
Residues in allowed regions	3.4

## Supplementary Materials

Supplementary Materials are available online.
