# Supplementary material for: Lectin PLL3, a Novel Monomeric Member of the Seven-Bladed β-Propeller Lectin Family
Source: Molecules. 2019 Dec 11;24(24):4540. doi: 10.3390/molecules24244540 (PMC6943638; doi:10.3390/molecules24244540)
Supplement: Supplementary file 1 [file molecules-24-04540-s001.zip › FileS1.docx]

**Lectin PLL3, a novel monomeric member of the seven‑bladed β‑propeller lectin family**

**Lukáš Faltinek**^1,†^**, Eva Fujdiarová**^2,3,†^**, Filip Melicher**^2,3^**, Josef Houser**^2,3^**, Martina Kašáková**^4^**, Nikolay Kondakov^5^, Leonid Kononov^5^, Kamil Parkan**^4^**, Sébastien Vidal**^6^**, Michaela Wimmerová**^1,2,3*^

^1^ Department of Biochemistry, Faculty of Science, Masaryk University, Kotlářská 2, 611 37 Brno, Czech Republic; faltinek@mail.muni.cz, michaw@chemi.muni.cz

^2^ Central European Institute of Technology, Masaryk University, Kamenice 5, 625 00 Brno, Czech Republic; eva.fujdiarova@ceitec.muni.cz, houser@mail.muni.cz, melicherfilip@mail.muni.cz

^3^ National Centre for Biomolecular Research, Faculty of Science, Masaryk University, Kotlářská 2, 611 37 Brno, Czech Republic

^4^ Department of Chemistry of Natural Compounds, University of Chemistry and Technology, Prague (UCTP), Technická 5, 166 28 Prague, Czech Republic; kasakovm@vscht.cz, parkank@vscht.cz

^5^ N.D. Zelinsky Institute of Organic Chemistry, Russian Academy of Sciences, Leninsky Prospect 47, 119 415 Moskow, Russia; leonid.kononov@gmail.com, nkondakov@gmail.com

^6^ Institut de Chimie et Biochimie Moléculaires et Supramoléculaires, CO2-Glyco, UMR 5246, CNRS, Université Claude Bernard Lyon 1, 43 Boulevard du 11 Novembre 1918, 6922, Villeurbanne, France; sebastien.vidal@univ-lyon1.fr

***** Correspondence: michaw@chemi.muni.cz

† These two authors contributed equally

Electronic Supplementary Information (ESI) available: [detail of any supplementary information available should be included here] See DOI: XXXXXX

**Supporting Information**

**Table of Contents**

1. **General Consideration**…………………………………………………………….....**2**
2. **Synthetic Procedures and Characterization Data**………………………………...**3**
3. **NMR Spectra**…………………………………………………………………………..**9**
4. **References**……………………………………………………………………………..**20**

### General Consideration

All reactions using anhydrous conditions were performed using flame-dried apparatus under an atmosphere of argon. Standard inert techniques were used in handling all air and moisture sensitive reagents. Anhydrous CH_2_Cl_2_ were obtained by distillation using CaH_2_ as a drying agent. All other solvents were used as supplied (Analytical or high-performance liquid chromatography grade), without prior purification. Reagents were purchased from various commercial suppliers and used as supplied, unless otherwise indicated. Brine refers to a saturated solution of sodium chloride. Anhydrous magnesium sulfate (MgSO_4_) was used as a drying agent after reaction workup, as indicated. Column chromatography was carried out using Material Harvest silica gel (pore size 60 Å, mesh 230−400 (40−63 μm)). Thin-layer chromatography (TLC) was carried out using Merck TLC Silica gel 60 F254 aluminum plates. Visualization of the TLC plates was achieved using a UV lamp Spektroline-ENF-240/F (Spectronics Corporation Westbury) (λ_max_ = 254 nm) and/or by spraying with cerium(IV) sulfate solution (1% in 10% H_2_SO_4_). Mobile phases are reported in relative composition (e.g., CH_2_Cl_2_/EtOAc 1:1 *v*/*v*). All ^1^H and ^13^C NMR spectra were recorded using a Bruker Avance III HD 600 (600 MHz for ^1^H; 150 MHz for ^13^C) spectrometer. ^1^H and ^13^C resonances were fully assigned using H,H-COSY, H,C-HSQC, and H,C-HMBC techniques. All chemical shifts are quoted on the δ scale in ppm and referenced using residual ^1^H solvent signal in ^1^H NMR spectra (δ(CHCl_3_) = 7.26 ppm, and ^13^C solvent signal in ^13^C NMR spectra (δ(CDCl_3_) = 77.0 ppm). Coupling constant (*J*) is reported in Hz with the following splitting abbreviations: s = singlet, d = doublet, t = triplet, q = quartet, and m = multiplet. High-resolution mass spectra were measured on a GC-MS Agilent 7200 Q-TOF (Agilent) spectrometer using electrospray ionization technique. Nominal and exact m/z values are reported in Daltons. Optical rotations were measured on an AUTOPOL IV (Rudolph Research Analytical,) polarimeter at temperature 25 °C and 589 nm sodium line with a path length *l* of 1.0 dm. Concentration c is given in g/100 mL. Specific rotation values are reported as a unitless number with implied units of (deg·mL)/(g·dm).

1. **Synthetic Procedures and Characterization Data**

### Scheme 1. Synthesis of *N*-(5-azido-3-oxa-pentyl)-2-(2,3,4-tri-*O*-acetyl-α-l-fucopyranosyl)ethanamide (2a).

### 2-(2,3,4-Tri-*O*-acetyl-α-l-fucopyranosyl)acetic acid (6). The 3-(tri-*O*-acetyl-α-l-fucopyranosyl)prop-1-ene 5 [47] (4.1 g, 11.5 mmol) was dissolved in CH_2_Cl_2_/CH_3_CN/H_2_O (33:33:49, *v*/*v*, 115 mL). NaIO_4_ (15.8 g, 68.82 mmol) and RuCl_3_ (71.4 mg, 344 µmol) were added and the reaction was stirred at rt. When the reaction was complete (~ 2 h, TLC silica gel, CH_2_Cl_2_/EtOAc = 4:1, *v*/*v*), the reaction mixture was partitioned between H_2_O (50 mL) and CH_2_Cl_2_ (100 mL). The aqueous phase was extracted CH_2_Cl_2_ (2 × 50 mL). The aqueous phase was then acidified to pH 4 by addition of conc. HCl and reextracted with CH_2_Cl_2_ (2 × 50 mL) and combined organic phases were washed with brine (100 mL), dried over MgSO_4_. After filtration and evaporation of the solvent, the residue was purified by flash column chromatography (silica gel, CH_2_Cl_2_/EtOAc 4:1 – 3:1, *v*/*v*) to afford title compound 6 (3.63 g, 84 %) as white foam. This compound was previously reported and our spectra match those described [48]. R*_f_* = 0.36 (silica gel, CH_2_Cl_2_/EtOAc = 4:1, *v*/*v*); $\left[ \boldsymbol{\alpha} \right]_{\mathbf{D}}^{\mathbf{25}}$ = - 85.4 (*c* 2.5, CHCl_3_); ^1^H NMR (CDCl_3_): δ (ppm): 5.39 (dd, 1H; *J* = 9.8, 5.6 Hz, H-2), 5.28 (dd, 1H; *J* = 3.4, 2.2 Hz, H-4), 5.18 (dd, 1H; *J* = 9.8, 3.4 Hz, H-3), 4.72 (dt, 1H; *J* = 9.0, 5.6 Hz, H-1), 4.06 (dq, 1H; *J* = 6.4, 2.0 Hz, H-5), 2.76 (dd, 1H; *J* = 15.5, 9.0 Hz, H-1´b), 2.66 (dd, 1H; *J* = 15.5, 5.6 Hz, H-1´a), 2.15, 2.04; 2.02 (3 × s, 9H; 3 × CH_3_CO), 1.17 (d, 3H; *J* = 6.2 Hz, H-6); ^13^C NMR (CDCl_3_): δ 175.4 (COOH), 170.5; 170.1, 169.7 (3 × CH_3_*C*O), 70.2 (C-4), 69.4 (C-1), 68.4 (C-3), 67.3 (C-2), 67.0 (C-5), 32.8 (C-1´), 20.7 (*C*H_3_CO), 15.8 (C-6); HRMS (ESI): *m/z* [M+H]^+^ calcd. for C_14_H_21_O_9_ 333.1107, found 333.1198.

### *N*-(5-azido-3-oxa-pentyl)-2-(2,3,4-tri-*O*-acetyl-α-l-fucopyranosyl)ethanamide (2a). To a solution of derivative 6 (368 mg, 1.11 mmol) in anhydrous CH_2_Cl_2_ (10 mL) were added HCTU (458 mg, 1.11 mmol) and DIPEA (0.193 mL, 1.11 mmol). After stirring of the reaction for 30 min at rt, a solution of 2-(2-azidoethoxy)ethanamin (360 mg, 2.77 mmol) in anhydrous CH_2_Cl_2_ (6 mL) and DIPEA (0.193 mL, 1.11 mmol) were added and the reaction mixture was stirred at rt. When the reaction was completed (~ 2 h, TLC silica gel, CH_2_Cl_2_/MeOH = 12:1, *v*/*v*), the reaction mixture was diluted with CH_2_Cl_2_ (10 mL) and extracted with 10% aqueous solution of HCl (2 × 40 mL), H_2_O (2 × 40 mL), saturated aqueous solution of NaHCO_3_ (2 × 40 mL) and brine (2 × 40 mL). The combined organic layer was dried over anhydrous MgSO_4_. After filtration and evaporation of solvent, the residue was purified by flash column chromatography (silica gel, CH_2_Cl_2_/MeOH 50/1, *v*/*v*) to afford title compound 2a (394 mg, 80 %) as colorless oil. R*_f_* = 0.75 (silica gel, CH_2_Cl_2_/MeOH = 12:1, *v*/*v*); $\left[ \boldsymbol{\alpha} \right]_{\mathbf{D}}^{\mathbf{25}}$ = -62.6 (*c* 1.6, CHCl_3_); ^1^H NMR (CDCl_3_): δ (ppm): 6.39 (br, 1H, NH), 5.31 (dd, 1H; *J* = 9.2, 5.2 Hz, H-2), 5.29 (dd, 1H; *J* = 3.2, 2.1 Hz, H-4), 5.16 (dd, 1H; *J* = 9.2, 3.2 Hz, H-3), 4.73-4.63 (m, 1H; H-1), 4.08 (dq, 1H; *J* = 6.5, 2.1 Hz, H-5), 3.68 (t, 2H; *J* = 4.7 Hz, CH_2_-D), 3.57 (t, 2H; *J* = 4.8 Hz, CH_2_-C), 3.52-3.45 (m, 2H; CH_2_-B), 3.39 (t, 2H; *J* = 4.7 Hz, CH_2-_E), 2.60 (dd, 1H; *J* = 15.5, 9.8 Hz, CH_2_-A), 2.45 (dd, 1H; *J* = 15.4, 4.2 Hz, CH_2_-A), 2.15, 2.06, 2.03 (3 × s, 3 × 3H, CH_3_CO), 1.21 (d, 3H; *J* = 6.5 Hz, H-6); ^13^C NMR (CDCl_3_): δ (ppm): 170.4, 170.0, 169.7 (CH_3_*C*O), 169.6 (CO-NH), 70.1 (CH_2_-D), 69.8 (C-4), 69.8 (CH_2_-C), 69.0 (C-1), 68.4 (C-3), 67.9 (C-2), 67.0 (C-5), 50.6 (CH_2_-E), 39.2 (CH_2_-B), 34.1 (CH_2_-A), 20.74, 20.73, 20.67 (*C*H_3_CO), 15.6 (C-6); HRMS (ESI): *m/z* [M+H]^+^ calcd. for C_18_H_29_N_4_O_9_ 445.1929, found 445.1925.

**Pillar[5]arene (3a).** A mixture of azide **2a** (283 mg, 639 µmol), (propargyl)_10_pillar[5]arene **1** [46] (52 mg, 53 µmol), CuSO_4_ˑ5H_2_O (1.3 mg, 5 µmol) and sodium l-ascorbate (3.2 mg, 16 µmol) in CH_2_Cl_2_/H_2_O (1:1, 4 mL) was vigorously stirred at room temperature under Ar. After 5 min, the pH value of reaction mixture was adjusted using PBS puffer (1 mL) to pH 10. The reaction was monitored by TLC (silica gel, CH_2_Cl_2_/MeOH 12:1, *v*/*v*) and after 96 h, the reaction mixture was diluted with H_2_O (20 mL) and the resulting solution was washed with CH_2_Cl_2_ (3 x 50 mL). The combined organic layers were washed with 1% aqueous solution of EDTA (20 mL) and dried over anhydrous MgSO_4_. After filtration and evaporation of solvent, the residue was dissolved in MeOH (4 mL) and Divergan^®^ (100 mg) was added. The suspension was stirred at rt. After 3 h, the suspension was filtered through a Celite^®^ pad, which was washed with MeOH (20 mL) and the filtrate was evaporated *in vacuo*. The residue was purified by flash column chromatography (silica gel, CH_2_Cl_2_/MeOH, linear gradient from 49:1 to 10:1, *v*/*v*) to afford **3a** (202 mg, 70 %) as a 1:1 mixture of diastereoisomers of colorless glassy foam. R*_f_*  = 0.47 (silica gel, CH_2_Cl_2_/MeOH = 12:1, *v*/*v*); ^1^H NMR (CDCl_3_), δ (ppm): 8.00 (br, 1H; triazolyl-H), 7.61 (br, 1H; NH), 6.88 and 6.86 (2 × brs, 1H; H_pillarene_-3), 5.40-5.31 (m, 1H; H-2), 5.29-5.17 (m, 2H; H-4, H-3), 5.04-4.80 (2 × m, 2H; CH_2F_), 4.77-4.68 (m, 1H; H-1), 4.64-4.48 (m, 2H; CH_2E_), 4.23-4.13 (m, 1H; H-5), 3.77-3.65 (m, 1H; H_bridge_), 3.60-3.48 (m, 2H; CH_2C_), 3.48-3.24 (m, 2H; CH_2B_), 2.86-2.74 and 2.57-2.46 (2 × m, 2H; CH_2A_), 2.16, 2.15, 2.02, 2.02, 2.00 and 2.00 (6 × s, 9H; CH_3_CO), 1.15-1.07 (m, 3H; H-6); ^13^C NMR (CDCl_3_), δ (ppm): 170.69, 170.68, 170.28, 170.24, 170.10, 169.74 and 169.73 (CH_3_*C*O and CO-NH), 149.76 (C1-_pillarene_),143.69 (C_triazol_), 129.02 (C2-_pillarene_), 124.60 (CH_triazol_), 116.05 (C3-_pillarene_), 70.50 (C-4), 69.98 (CH_2C_), 69.91 (C-1), 68.93 (CH_2D_), 68.57 (C-3), 67.65 (C-2), 66.47 (C-5), 62.19 (CH_2F_), 50.36 (CH_2E_), 39.10 (CH_2B_), 33.89 (CH_2A_), 29.40 (CH_2bridge_), 20.78, 20.76, 20.69 (*C*H_3_CO), 15.96 (C-6); HRMS (ESI): *m/z* [M+2Na]^2+^ calcd. for C_245_H_330_N_40_O_100_Na_2_ 2740.5926, found 2740.5997.

**Pillar[5]arene (4a)**. To a solution of compound **3a** (157 mg, 29 µmol) in MeOH/H_2_O (2:0.5 mL) was dropwise added Et_3_N (0.5 mL, 3.8 mmol) and the reaction mixture was stirred at rt for 16 h. Then the reaction was concentrated, and the residue was purified by flash column chromatography (C8 functionalized silica gel, H_2_O/MeOH linear gradient from 90:10 to 70:30) to afford title compound **4a** (118 mg, 98 %) as a 1:1 mixture of diastereoisomers of colorless solid. ^1^H NMR (D_2_O), δ (ppm): 8.25-7.53 (m, 1H; triazolyl-H), 6.61 and 6.57 (2 × brs, 1H; H_pillarene_-3), 4.73-4.63 (m, 2H; CH_2F_), 4.59-4.35 (m, 2H; CH_2E_), 4.35-4.26 (m, 1H; H-1), 3.94-3.87 (m, 1H; H-2), 3.86-3.69 (m, 2H; H-5), 3.68-3.61 (m, 1H; H-4), 3.61-3.53 (m, 1H; H-3), 3.55 (overlapped m, 1H; H_bridge_), 3.49-3.41 (m, 2H; CH_2C_), 3.27-3.07 (m, 2H; CH_2B_), 2.54-2.45 and 2.45-2.36 (2 × m, 2H; CH_2A_), 1.01 (d, *J* =6.2 Hz, 3H; H-6); ^13^C NMR (D_2_O), δ (ppm): 173.55 and 173.52 (CO-NH), 149.97 (C1-_pillarene_),143.58 and 143.49 (C_triazol_), 129.04 (C2-_pillarene_), 125.23and 125.154 (CH_triazol_), 117.00 (C3-_pillarene_), 73.37 (C-1), 71.50 (C-4), 69.89 (C-3), 68.82 (CH_2C_), 68.50 (CH_2D_), 67.76 (C-5), 67.21 and 67.19 (C-2), 61.95 (CH_2F_), 50.05 (CH_2E_), 38.92 (CH_2B_), cca 31 (broad CH_2bridge_), 32.39 and 32.37 (CH_2A_), 15.72 (C-6); HRMS (ESI): *m/z* [M+3Na]^3+^ calcd. for C_185_H_270_N_40_O_70_Na 1413.9503, found 1413.9503; *m/z* [M+2Na]^2+^ calcd. for C_185_H_270_N_40_O_70_Na 2110.4341, found 2110.4338.

**Pillar[5]arene (3b).** A mixture of azide **2b** [39] (223 mg, 651 µmol), pillar[5]arene **1** [46] (53 mg, 54 µmol), CuSO_4_ˑ5H_2_O (1.4 mg, 5 µmol) and sodium l-ascorbate (3.2 mg, 16 µmol) in CH_2_Cl_2_/H_2_O (1:1, 4 mL) was vigorously stirred at room temperature under Ar. After 5 min, the pH value of reaction mixture was adjusted using PSB puffer (1 mL) to pH 10. The reaction was monitored by TLC (silica gel, CH_2_Cl_2_/MeOH 15:1, *v*/*v*) and after 96 h, the reaction mixture was diluted with H_2_O (20 mL) and the resulting solution was washed with CH_2_Cl_2_ (3 x 50 mL). The combined organic layers were washed with 1% aqueous solution of EDTA (15 mL) and dried over anhydrous MgSO_4_. After filtration and evaporation of solvent, the residue was dissolved in MeOH (4 mL) and Divergan^®^ (100 mg) was added. The suspension was stirred at rt. After 3 h, the suspension was filtered through a Celite^®^ pad, which was washed with MeOH (20 mL) and the filtrate was evaporated *in vacuo*. The residue was purified by flash column chromatography (silica gel, CH_2_Cl_2_/MeOH, linear gradient from 100:1 to 50:1, *v*/*v*) to afford **3b** (204 mg, 85 %) as a 1:1 mixture of diastereoisomers of colorless glassy foam. R*_f_* = 0.49 (silica gel, CH_2_Cl_2_/MeOH = 19:1, *v*/*v*); ^1^H NMR (CDCl_3_), δ (ppm): 7.97-7.82 (brm, 1H; triazolyl-H), 6.97-6.75 (m, 1H; H_pillarene_-3), 5.33-5.08 (m, 3H; H-2, H-3, H-4), 5.00-4.80 (m, 2H; CH_2C_), 4.60-4.34 (m, 2H; CH_2B_), 4.31-4.16 (m, 1H; H-1), 4.13-3.97 (m, 1H; H-5), 3.84-3.66 (m, 1H; H_bridge_), 2.58-2.35 and 2.22-2.06 (2 × m, 2H; CH_2A_), 2.12, 2.00 and 1.95 (3 × s, 9H; CH_3_CO), 1.19-1.05 (m, 3H; H-6); ^13^C NMR (CDCl_3_), δ (ppm): 170.44, 169.90 and 169.78 (CH_3_*C*O), 149.53 and 149.46 (C1-_pillarene_), 144.31 and 144.27 (C_triazol_), 128.58 and 128.39 (C2-_pillarene_), 123.43 (CH_triazol_), 115.48 and 115.10 (C3-_pillarene_), 70.46 (C-4), 70.25 (C-1), 68.30 (C-3), 67.72 (C-2), 65.98 (C-5), 62.49 and 62.32 (CH_2C_), 47.24 (CH_2B_), 29.64 (CH_2bridge_), 26.29 and 26.04 (CH_2A_), 20.76, 20.64 and 20.62 (*C*H_3_CO), 15.94 (C-6); HRMS (ESI): *m/z* [M+3H]^3+^ calcd. for C_205_H_262_N_30_O_80_ 1474.9139, found 1474.9095; *m/z* [M+2H]^2+^ calcd. for C_205_H_262_N_30_O_80_ 221.8672, found 2211.8595.

**Pillar[5]arene (4b).** To a solution of compound **3b** (245 mg, 29 µmol) in MeOH/H_2_O (2:0.5 mL) was dropwise added Et_3_N (0.5 mL, 3.8 mmol) and the reaction mixture was stirred at rt for 16 h. Then the reaction was concentrated, and the residue was purified by flash column chromatography (C8 functionalized silica gel, H_2_O/MeOH linear gradient from 80:20 to 50:50) to afford title compound **4b** (164 mg, 94 %) as a 1:1 mixture of diastereoisomers of colorless solid. ^1^H NMR (D_2_O), δ (ppm): 7.58 and 7.54 (2 × brs, 1H; triazolyl-H), 6.37 (s, 1H; H_pillarene_-3), 4.57 and 4.55 (2 × brs, 1H; CH_2C_), 4.41-4.23 (m, 2H; CH_2B_), 4.19 and 4.18 (1 × brs, 1H; CH_2C_), 3.93-3.80 (m, 2H; H-1, H-2), 3.67-3.48 (m, 3H; H-3, H-4, H-5), 3.55 (overlapped m, 1H; H_bridge_), 2.26-1.99 (m, 2H; CH_2A_), 0.97 and 0.95 (2 × d, 3H; *J* =5.6 Hz, H-6); ^13^C NMR (D_2_O), δ (ppm): 150.31 and 150.17 (C1-_pillarene_),143.22 and 143.17 (C_triazol_), 128.95 and 128.79 (C2-_pillarene_), 125.02 and 124.80 (CH_triazol_), 116.88 and 116.69 (C3-_pillarene_), 73.39 (C-1), 71.50 and 71.44 (C-4), 69.90 and 69.86 (C-3), 67.35 (C-2 and C-5), 61.07 and 61.92 (CH_2C_), 47.83 and 47.73 (CH_2B_), 31.49 (CH_2bridge_), 24.76 and 24.66 (CH_2A_), 15.71 and 15.67 (C-6); HRMS (ESI): *m/z* [M+3H]^3+^ calcd. for C_145_H_203_N_30_O_50_ 1554.8083, found 1554.8109; *m/z* [M+2H]^2+^ calcd. for C_145_H_202_N_30_O_50_ 1581.7088, found 1581.7078; *m/z* [M+H+Na]^+^ calcd. for C_145_H_201_N_30_O_50_Na 1592.6997, found 1592.7020.

1. **NMR Spectra**

**Pillar[5]arene (3a).**

**Figure 1. ^1^H NMR spectrum (600 MHz, CDCl_3_) of pillar[5]arene (3a).**


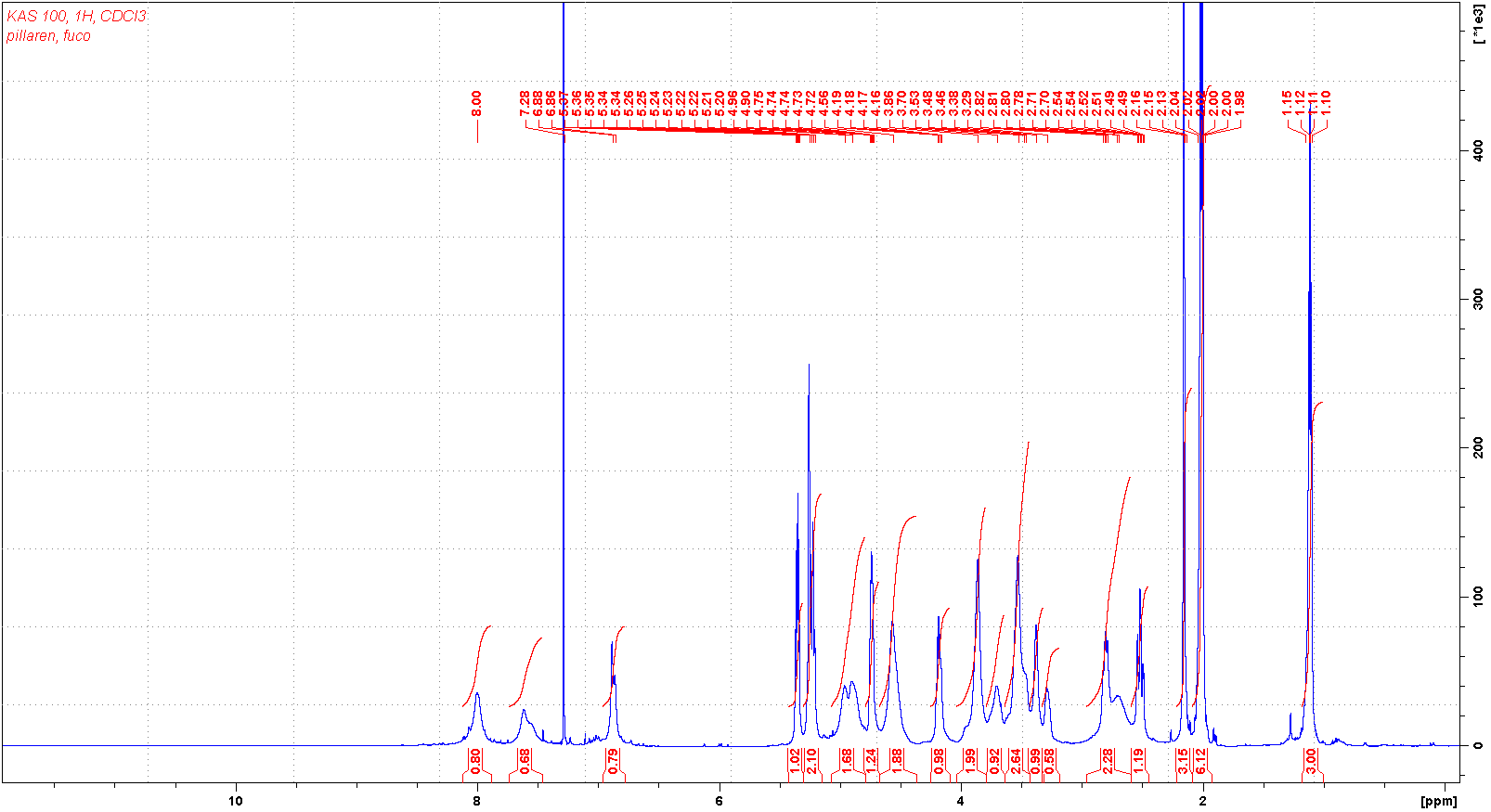


**Figure 2. ^13^C NMR spectrum (150 MHz, CDCl_3_) of pillar[5]arene (3a).**


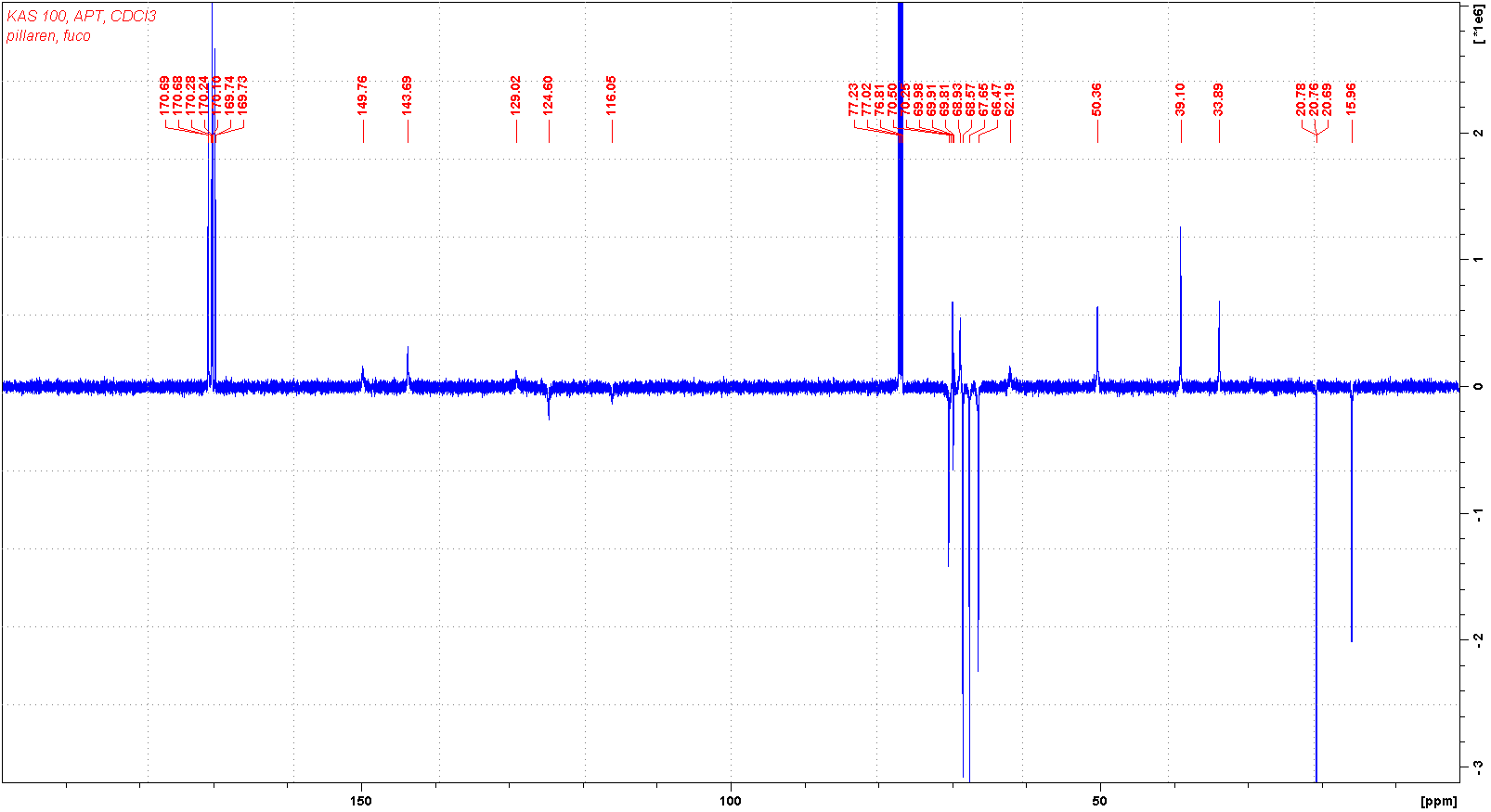


**Figure 3. COSY spectrum (CDCl_3_) of pillar[5]arene (3a).**


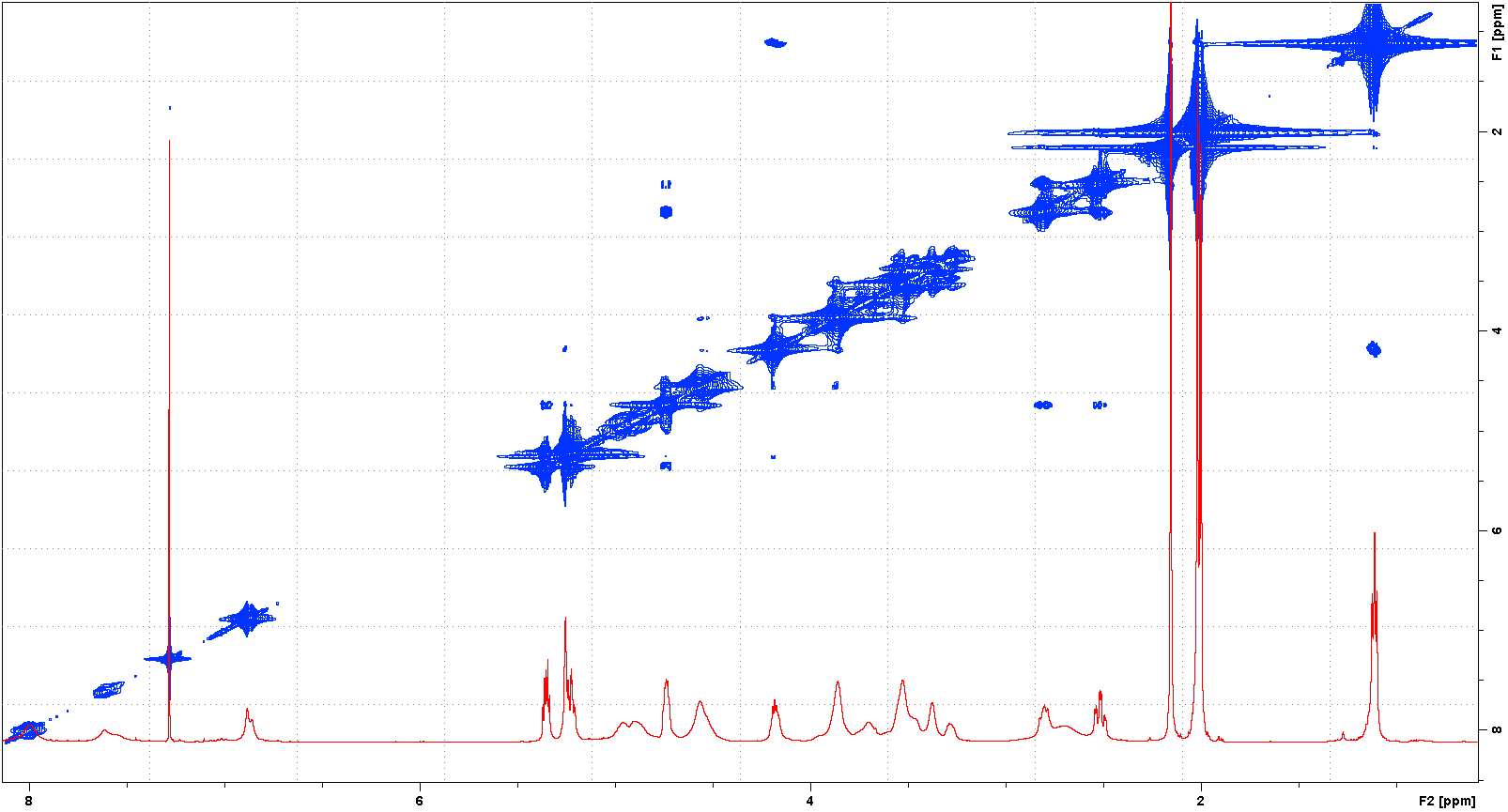


**Figure 4. HMQC spectrum (CDCl_3_) of pillar[5]arene (3a).**


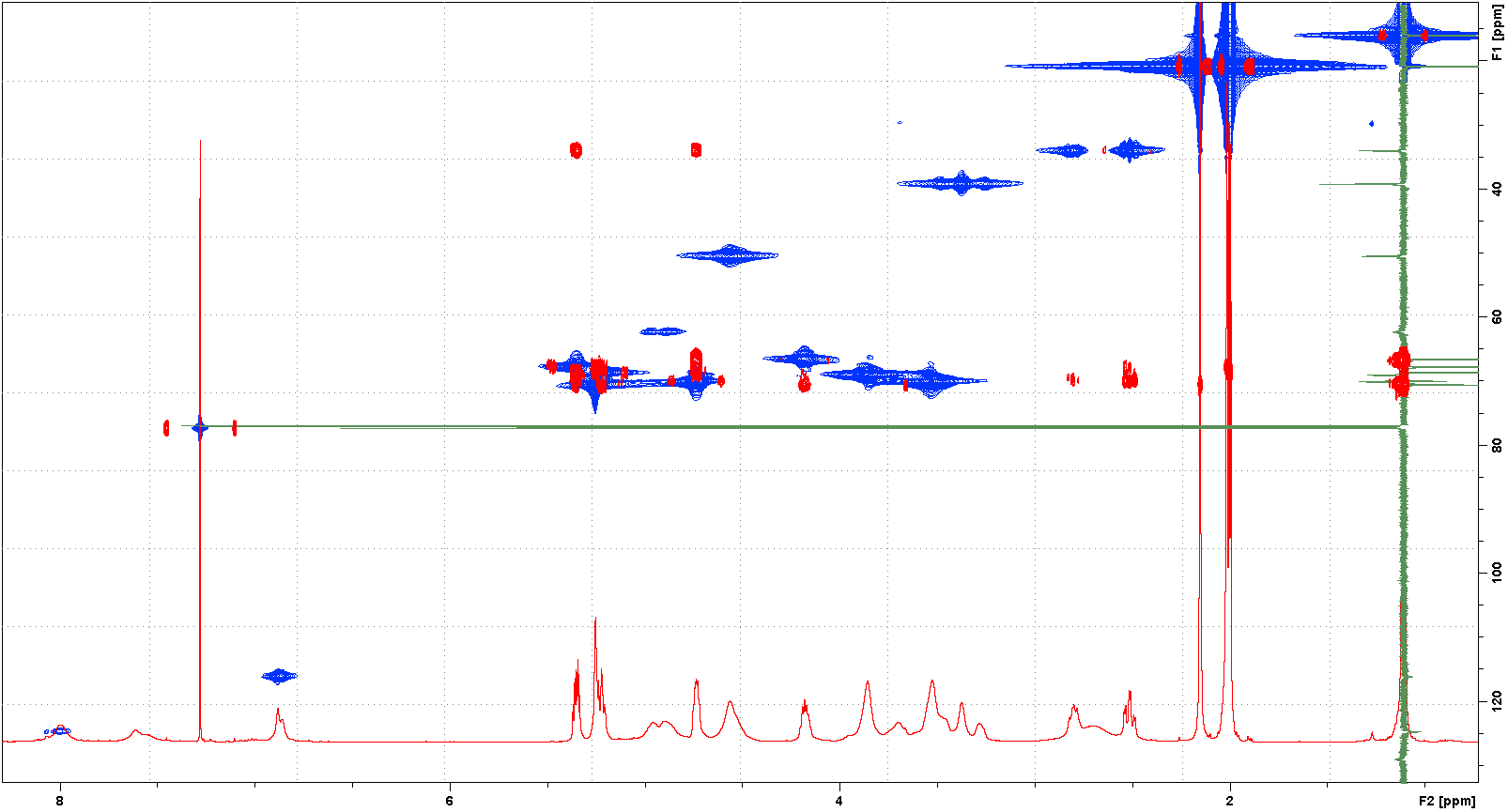


**Pillar[5]arene (4a).**

**Figure 5. ^1^H NMR spectrum (600 MHz, D_2_O) of pillar[5]arene (4a).**


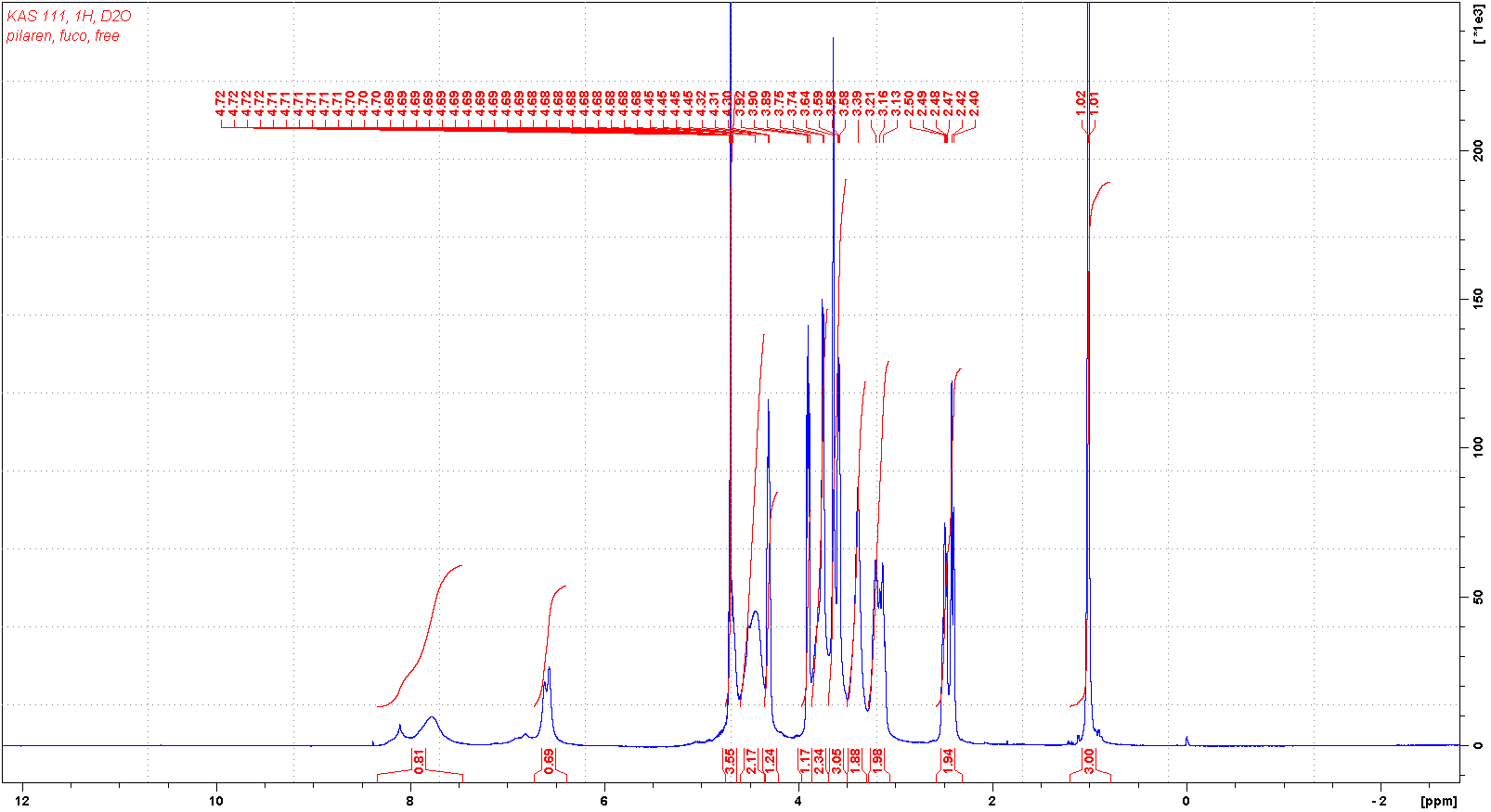


**Figure 6. ^13^C NMR spectrum (150 MHz, D_2_O) of pillar[5]arene (4a).**


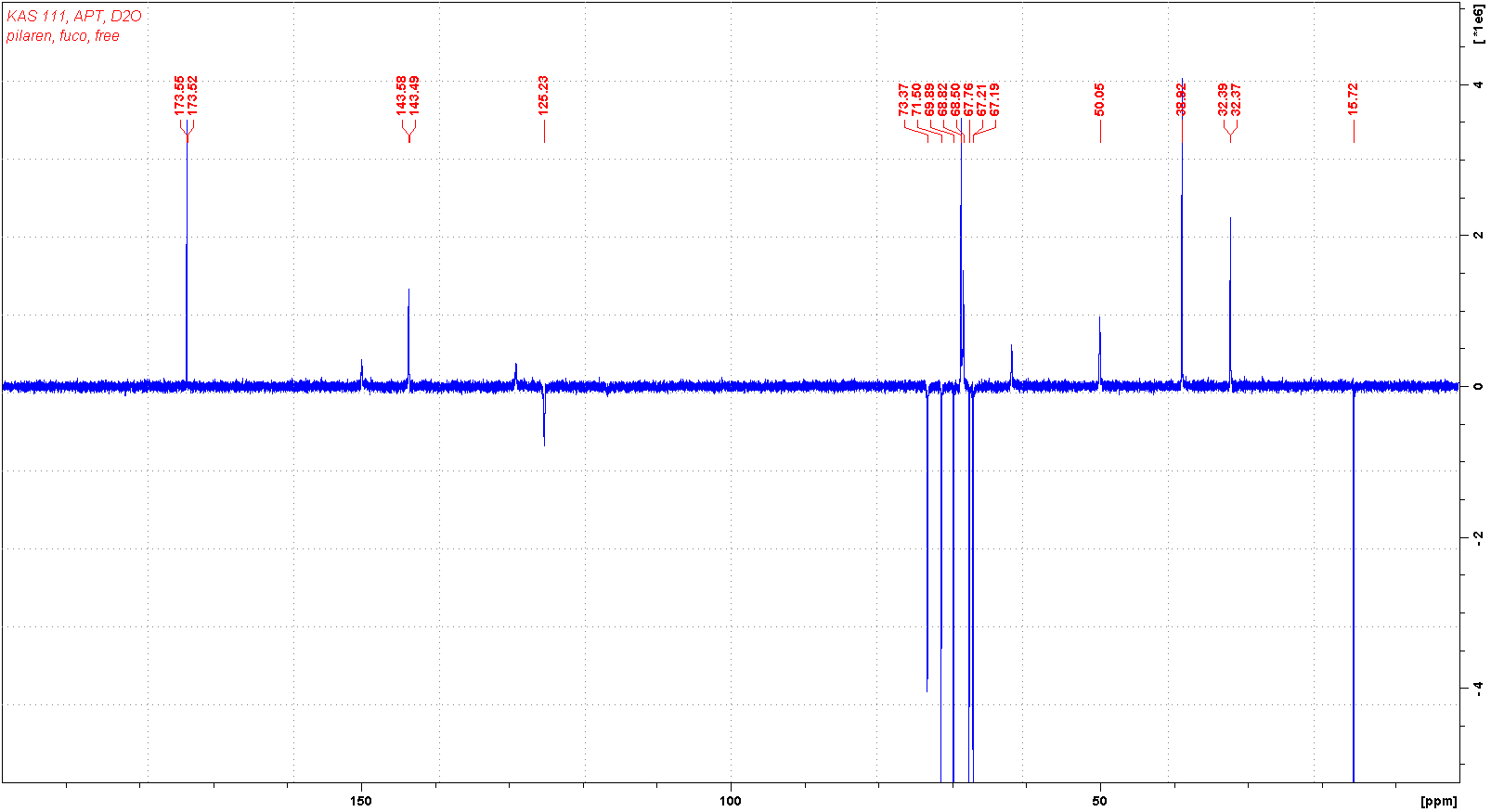


**Figure 7. COSY spectrum (D_2_O) of pillar[5]arene (4a).**


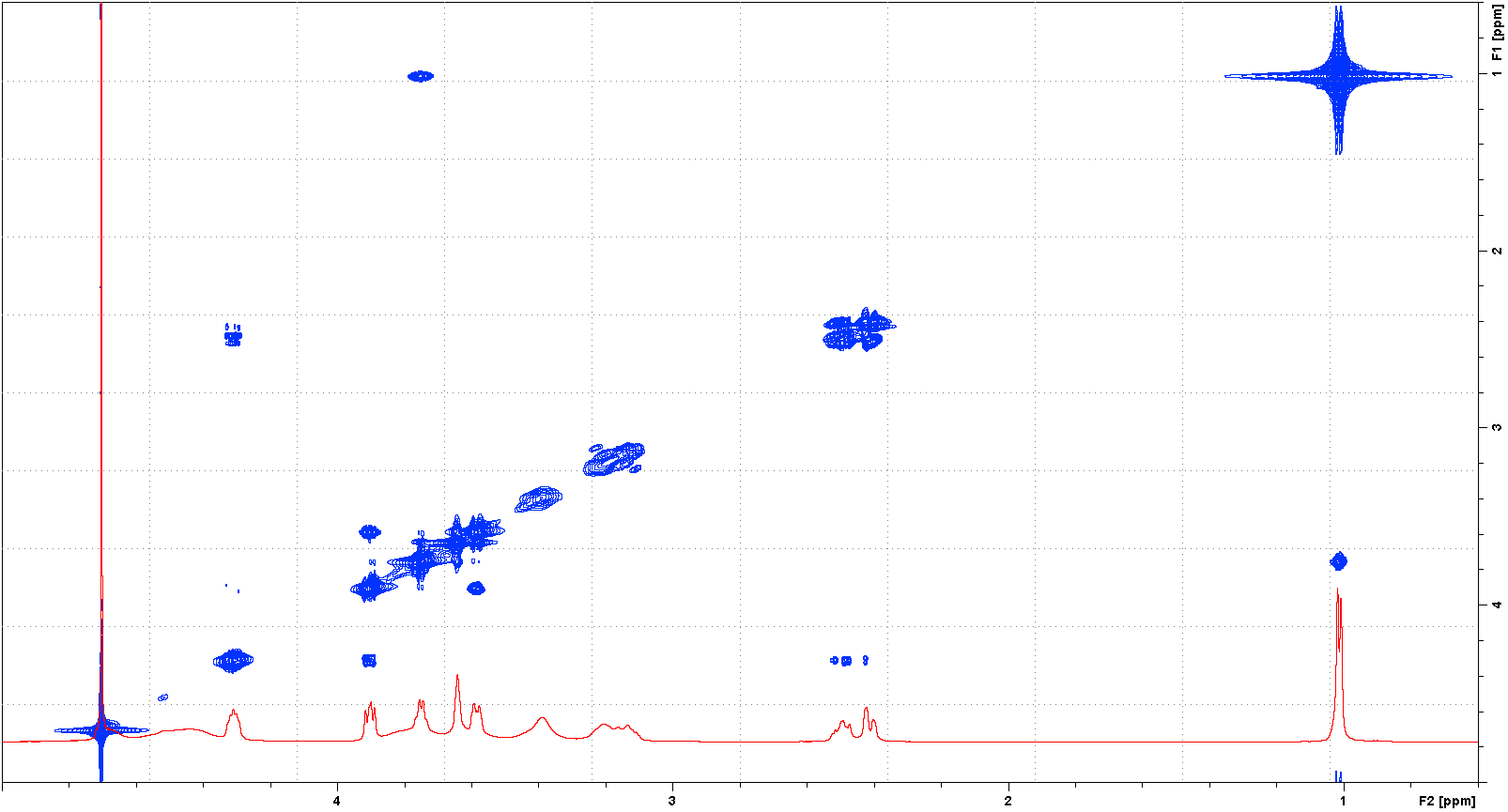


**Figure 8. HMQC spectrum (D_2_O) of pillar[5]arene (4a).**


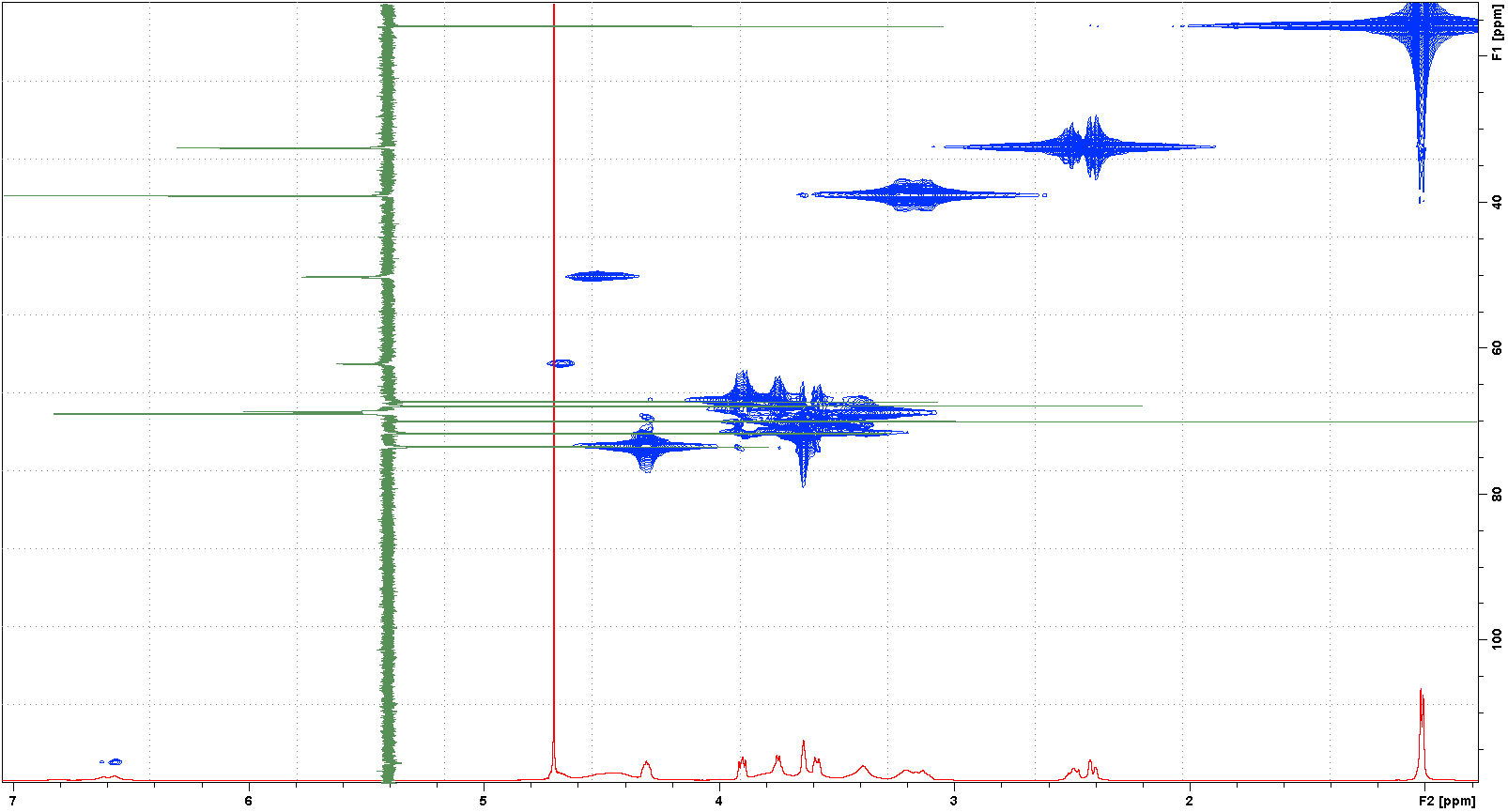


**Pillar[5]arene (3b).**

**Figure 9. ^1^H NMR spectrum (600 MHz, CDCl_3_) of pillar[5]arene (3b).**


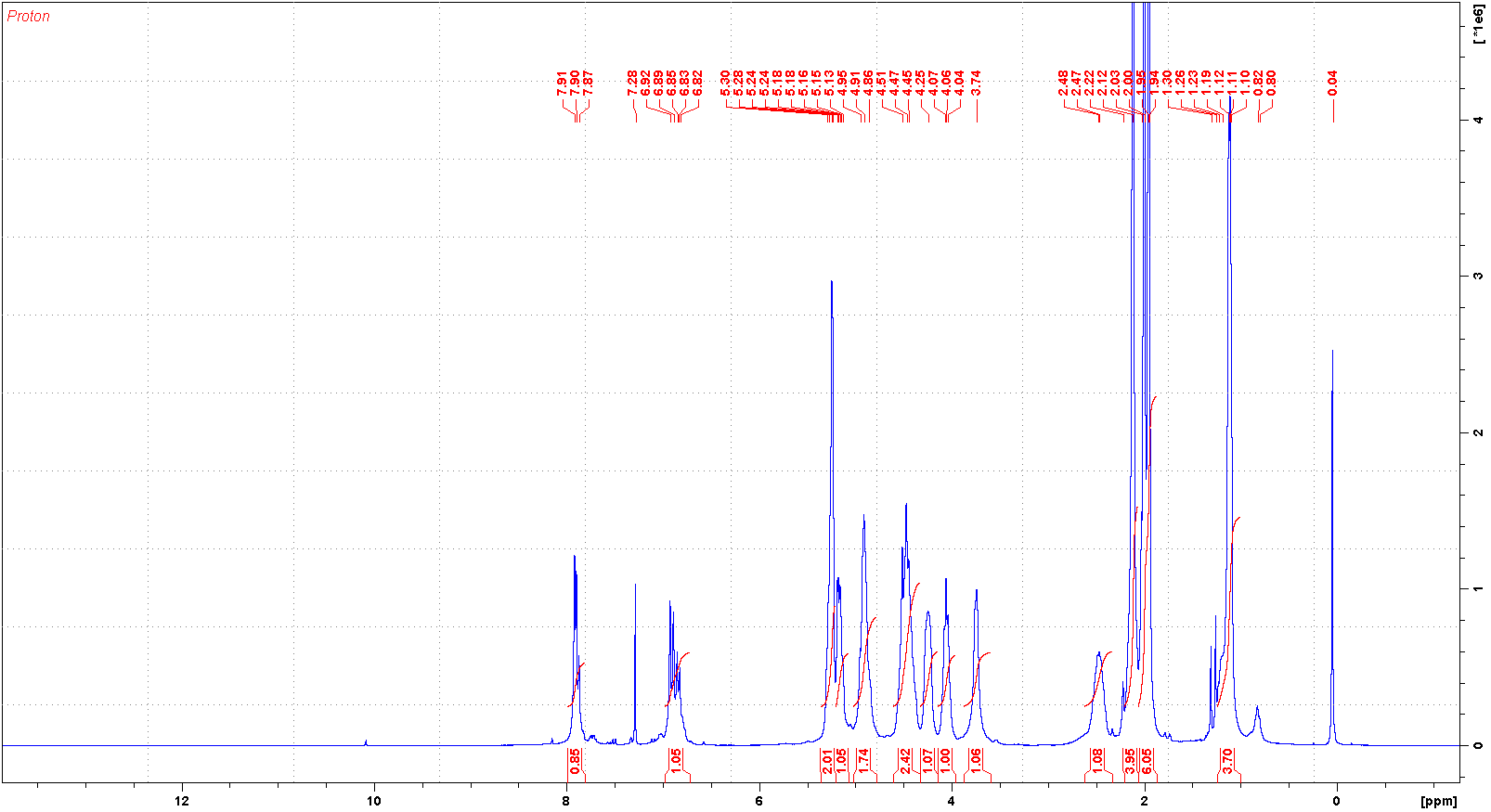


**Figure 10. ^13^C NMR spectrum (150 MHz, CDCl_3_) of compound (3b).**


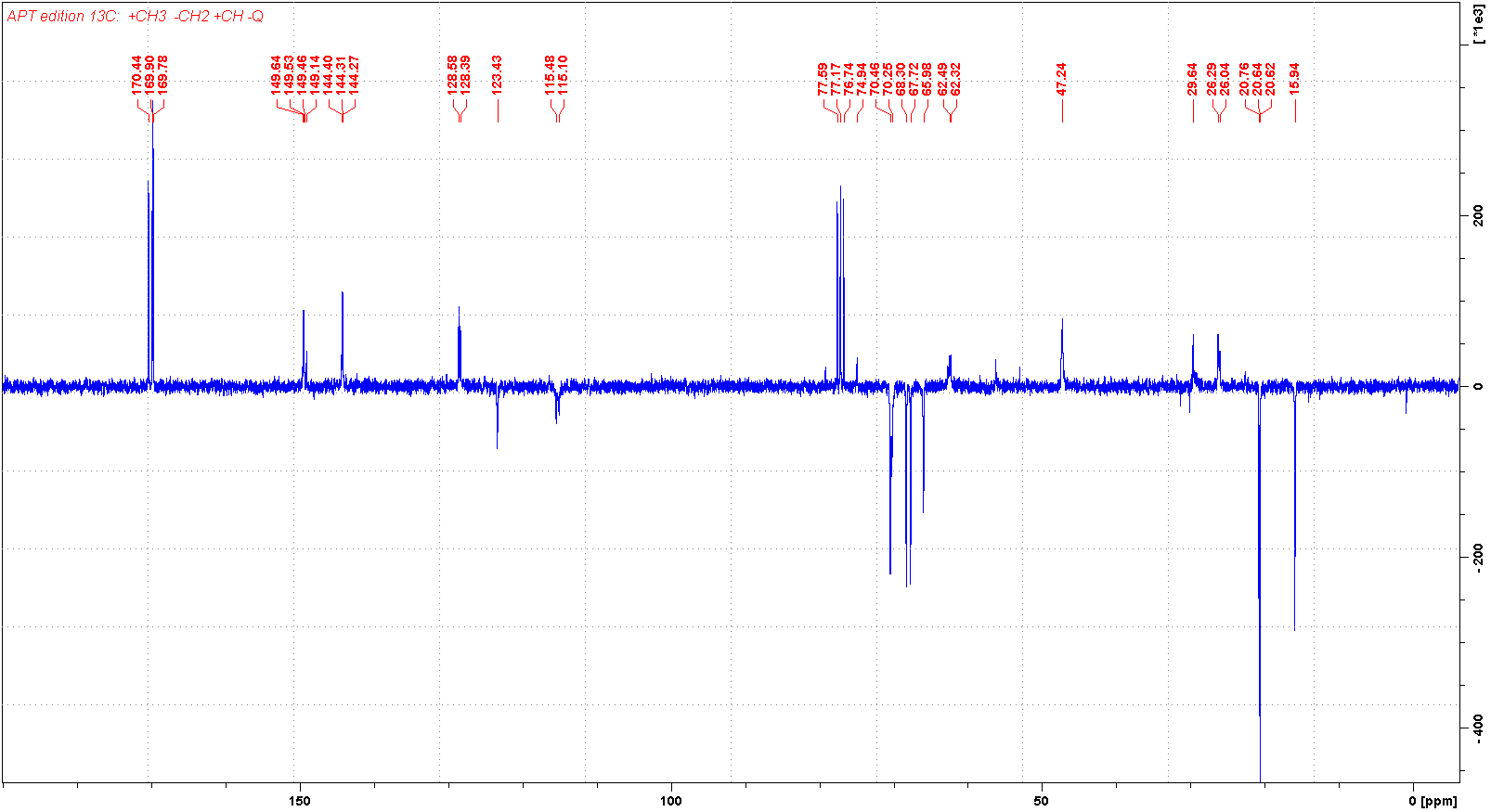


**Figure 11. COSY spectrum (CDCl_3_) of pillar[5]arene (3b).**


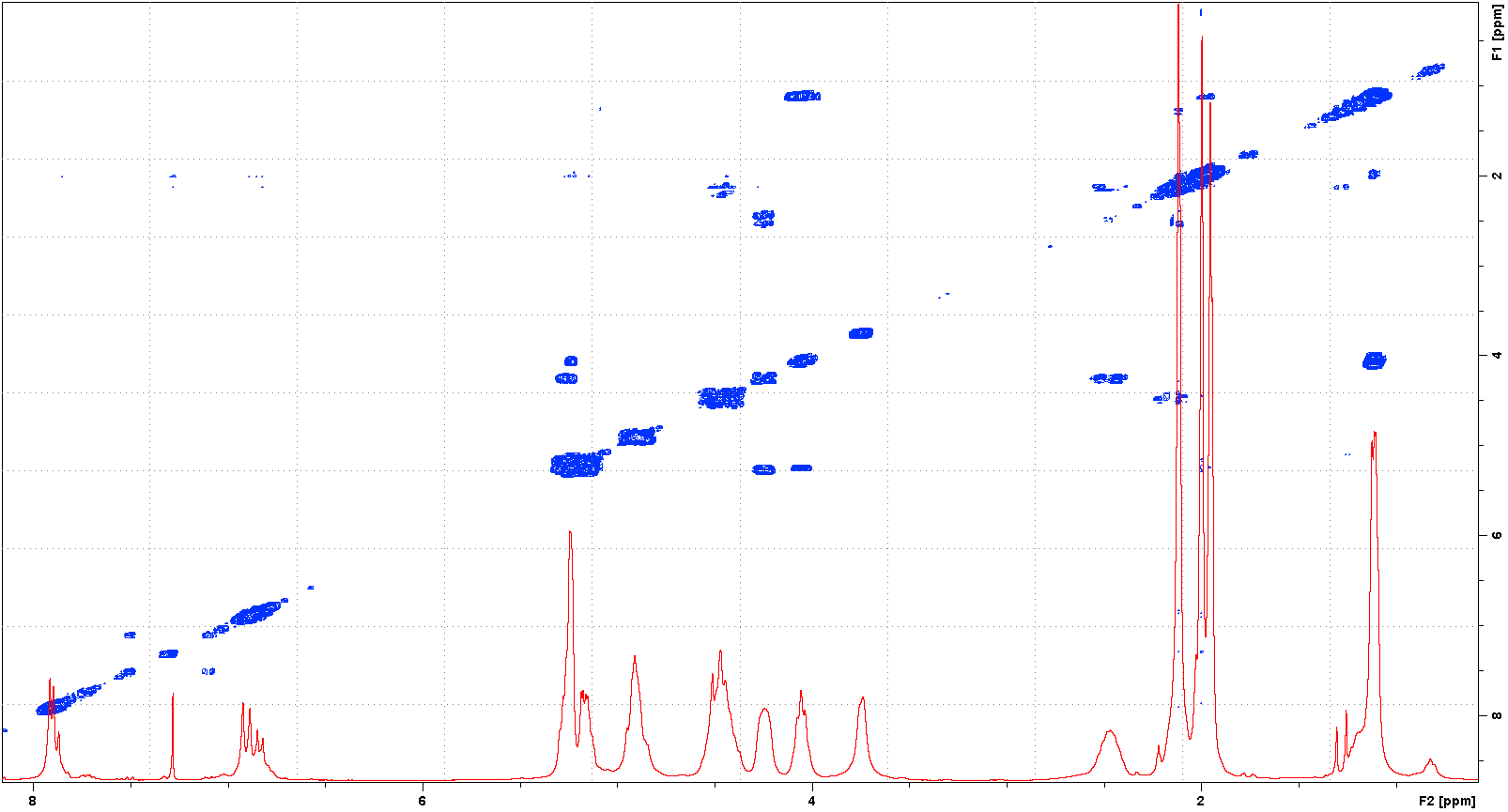


**Figure 12. HMQC spectrum (CDCl_3_) of pillar[5]arene (3b).**


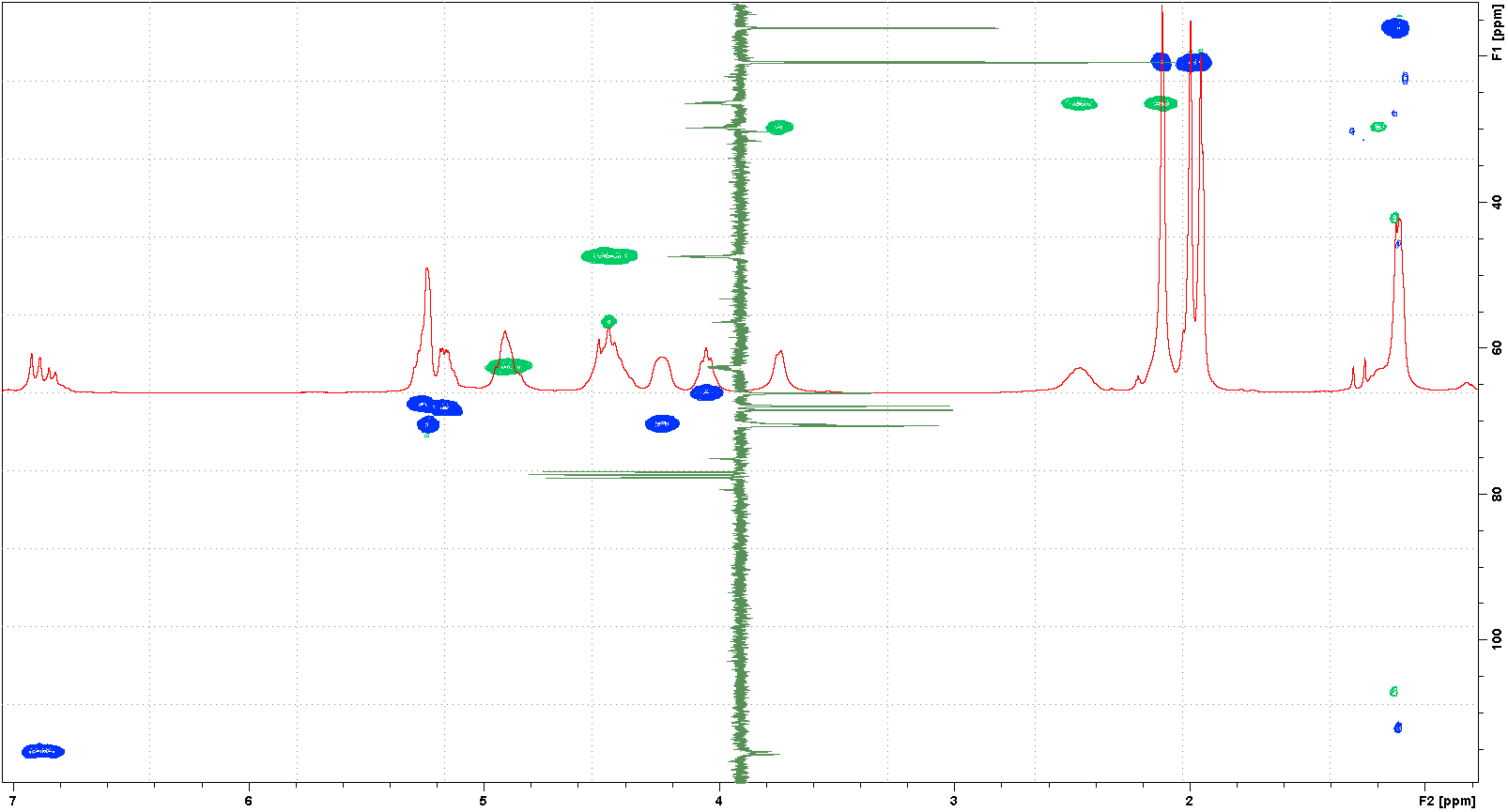


**Pillar[5]arene (4b).**

**Figure 13. ^1^H NMR spectrum (600 MHz, D_2_O) of pillar[5]arene (4b).**


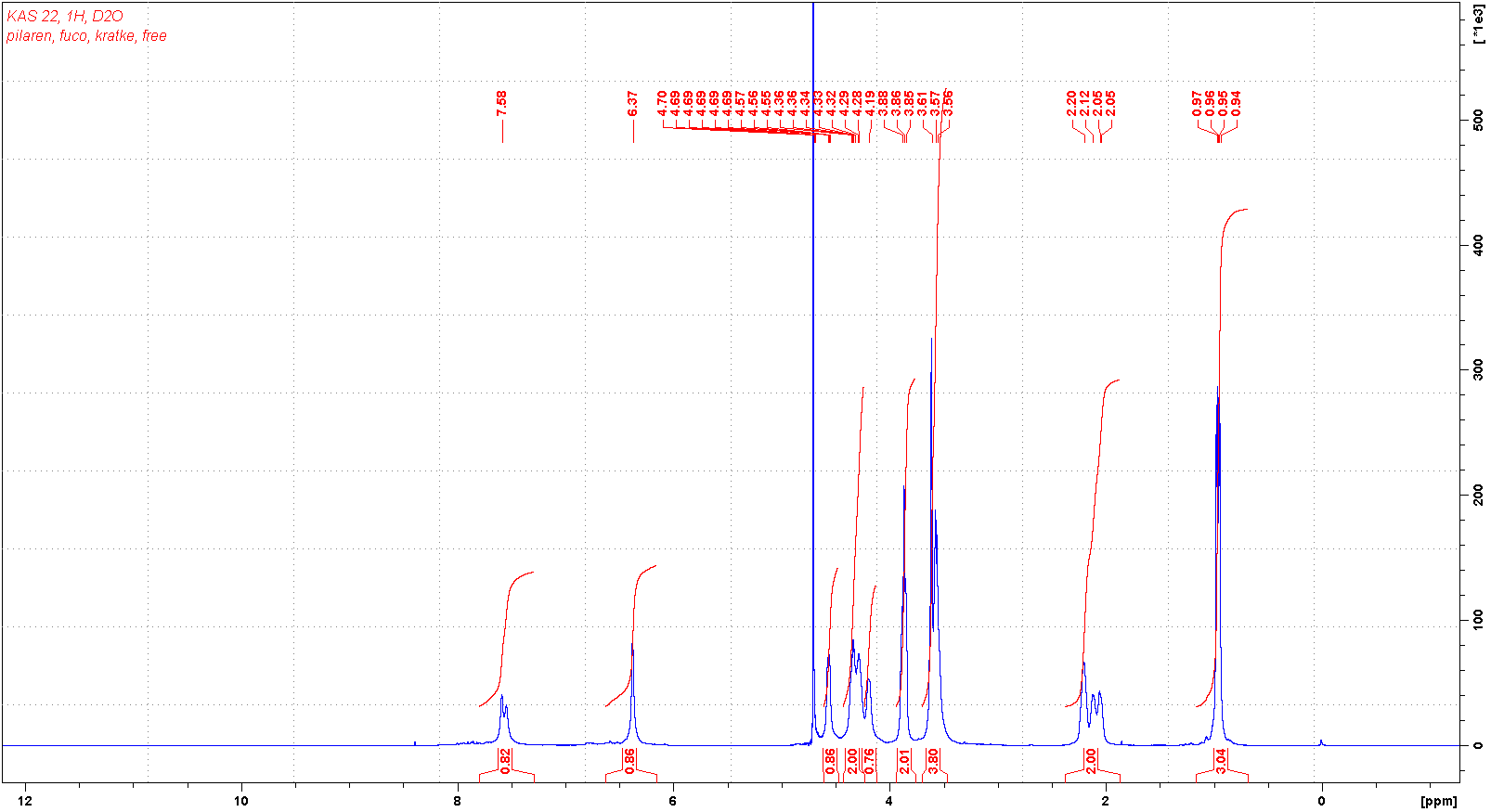


**Figure 14. ^13^C NMR spectrum (150 MHz, D_2_O) of pillar[5]arene (4b).**


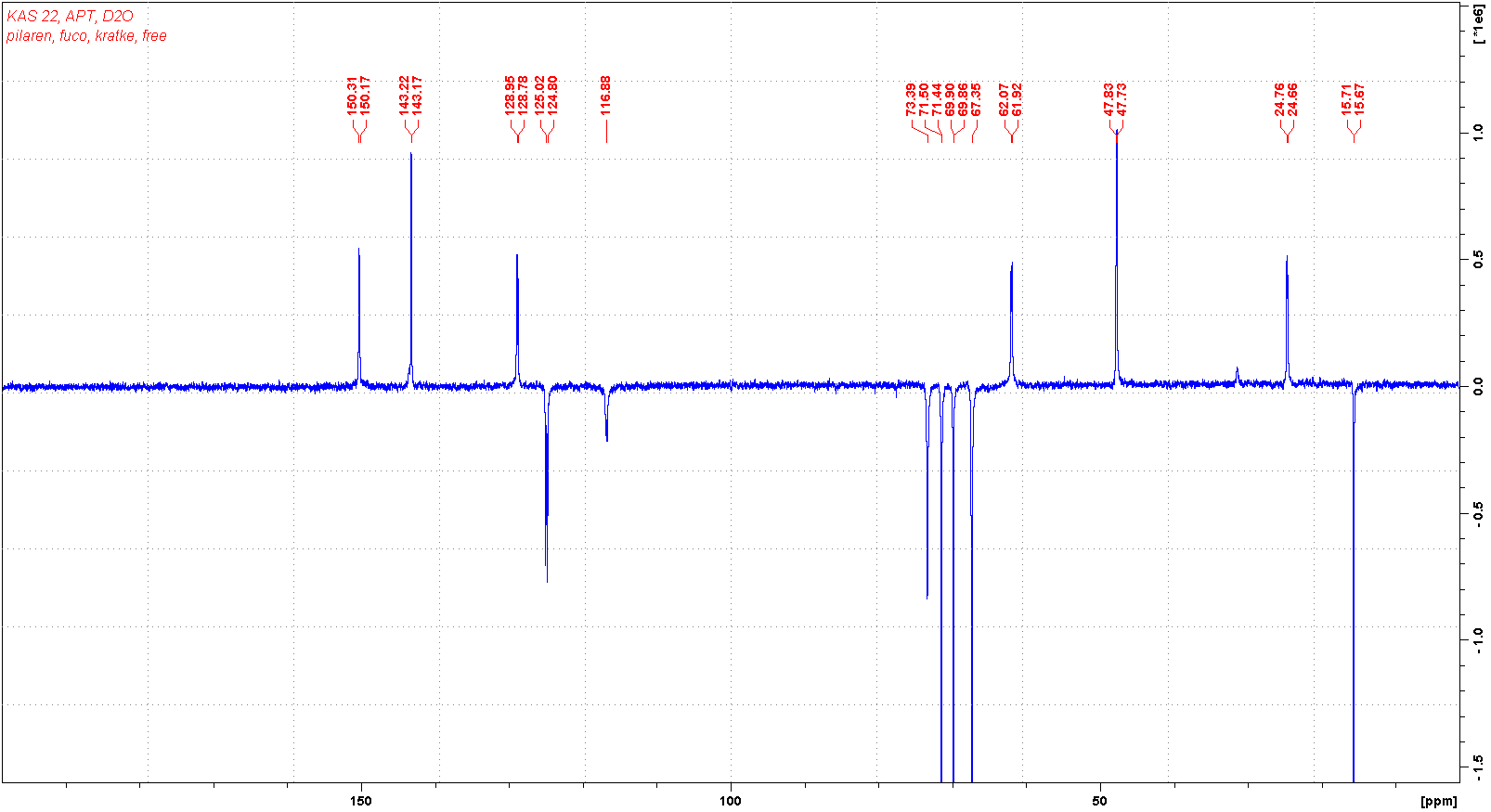


**Figure 15. COSY spectrum (D_2_O) of pillar[5]arene (4b).**


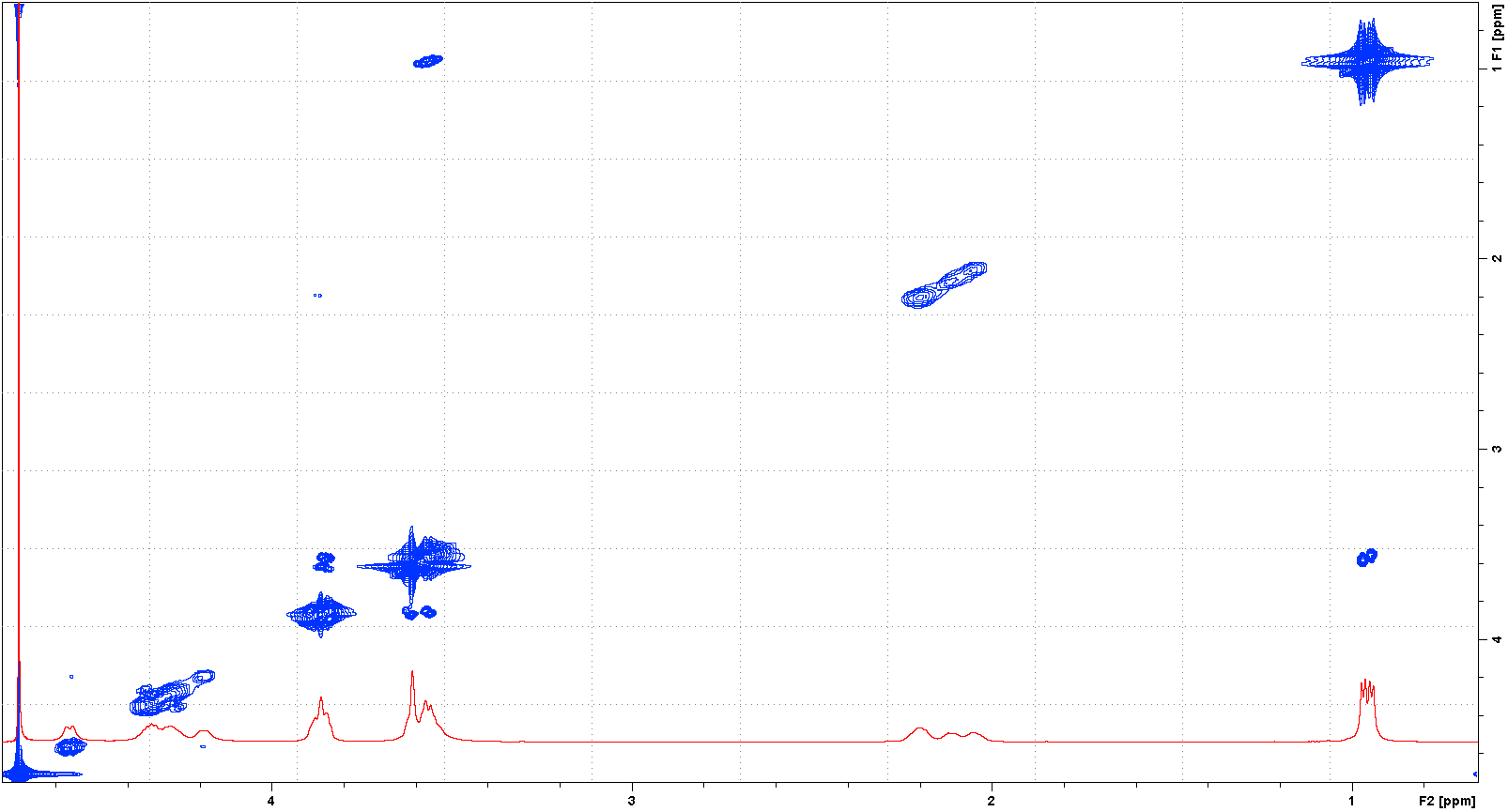


**Figure 16. HMQC spectrum (D_2_O) of pillar[5]arene (4b).**


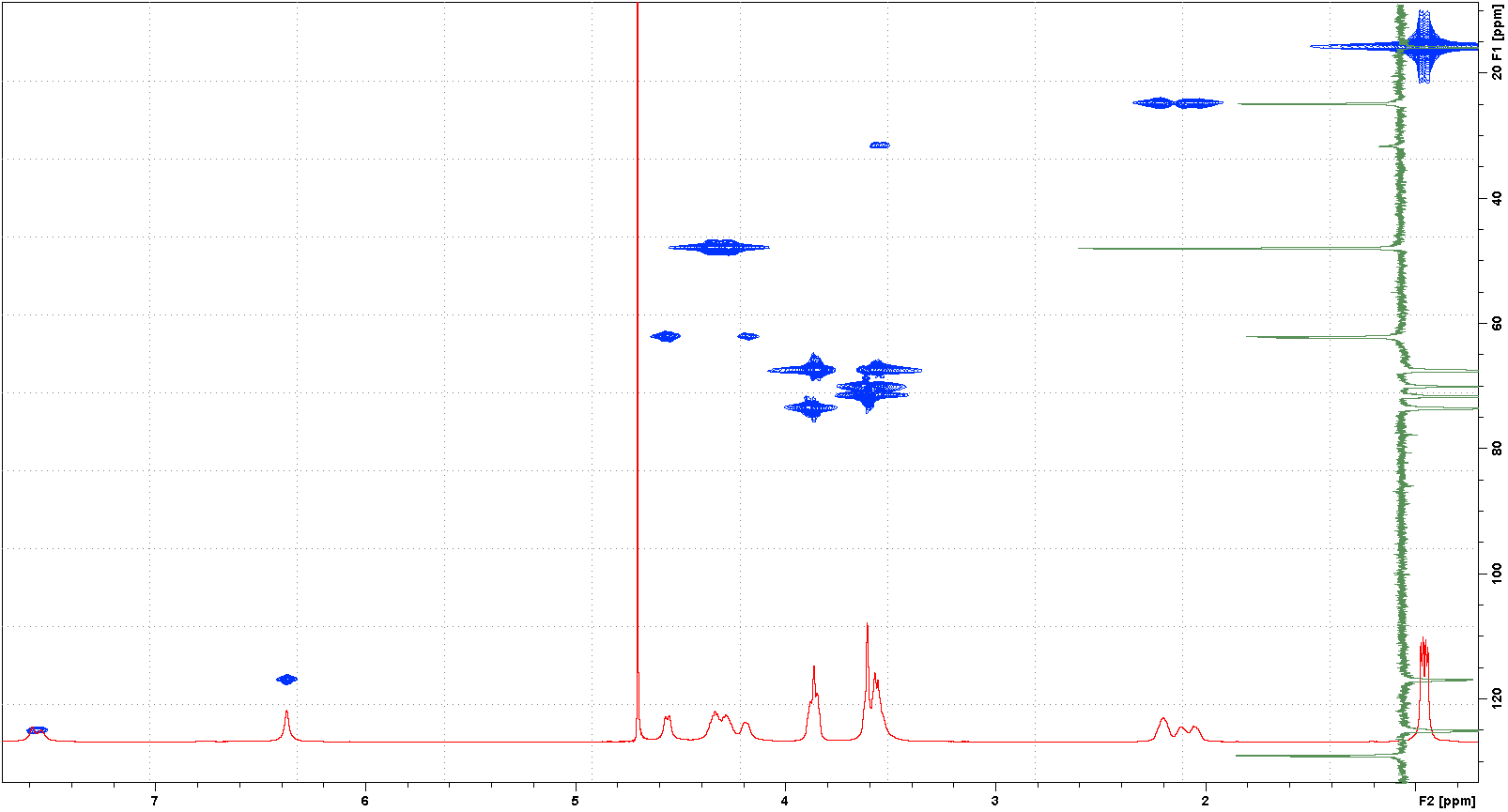


1. References

[39] Bertolotti, B.; Sutkeviciute, I.; Ambrosini, M.; Ribeiro-Viana, R.; Rojo, J.; Fieschi, F.; Dvořáková, H.; Kašáková, M.; Parkan, K.; Hlaváčková, M.; et al. Polyvalent C-glycomimetics based on l-fucose or d-mannose as potent DC-SIGN antagonists. *Org. Biomol. Chem.* 2017, *15*, 3995–4004.

[46] Nierengarten, I.; Nothisen, M.; Sigwalt, D.; Biellmann, T.; Holler, M.; Remy, J.-S.; Nierengarten, J.-F. Polycationic Pillar[5]arene Derivatives: Interaction with DNA and Biological Applications. *Chem. Eur. J.* 2013, *19*, 17552–17558.

[47] Kašáková, M.; Bertolotti, B.; Dong, L.; Rousset, A.; Kánya, N.; Moravcová, J.; Vidal, S. 3-(2,3,4-Tri-*O*-acetyl-α-l-fucopyranosyl)-prop-1-ene. In Carbohydrate Chemistry: Proven Synthetic Methods, Kosma, P., Ed. CRC Press, 2020; Vol. 5 (in press).

[48] Kolomiets, E.; Johansson, E.M.V.; Renaudet, O.; Darbre, T.; Reymond, J.-L. Neoglycopeptide Dendrimer Libraries as a Source of Lectin Binding Ligands. *Org. Lett.* 2007, *9*, 1465–1468.
