# Supplementary material for: Lectin PLL3, a Novel Monomeric Member of the Seven-Bladed β-Propeller Lectin Family
Source: Molecules. 2019 Dec 11;24(24):4540. doi: 10.3390/molecules24244540 (PMC6943638; doi:10.3390/molecules24244540)
Supplement: Supplementary file 1 [file molecules-24-04540-s001.zip › Supplementary_Materials.docx]

## **Supplementary Materials**

**Table S1. Glycan array - Raw data**.

(XLSX)

**Table S2. Glycan array results**. Screening of glycans printed on a microarray chip (Semiotik, Moscow; Slide number 10085636) via a standard manufacturer’s procedure. Results were calculated from hexaplicates.

(XLSX)

**File S1. Synthesis of pillar[5]arene-based glycoclusters.** Synthetic procedures and characterization data of compounds **2a-4a** and **2b-4b** including NMR spectra of compounds **3a-4a** and **3b-4b**.

(DOCX)


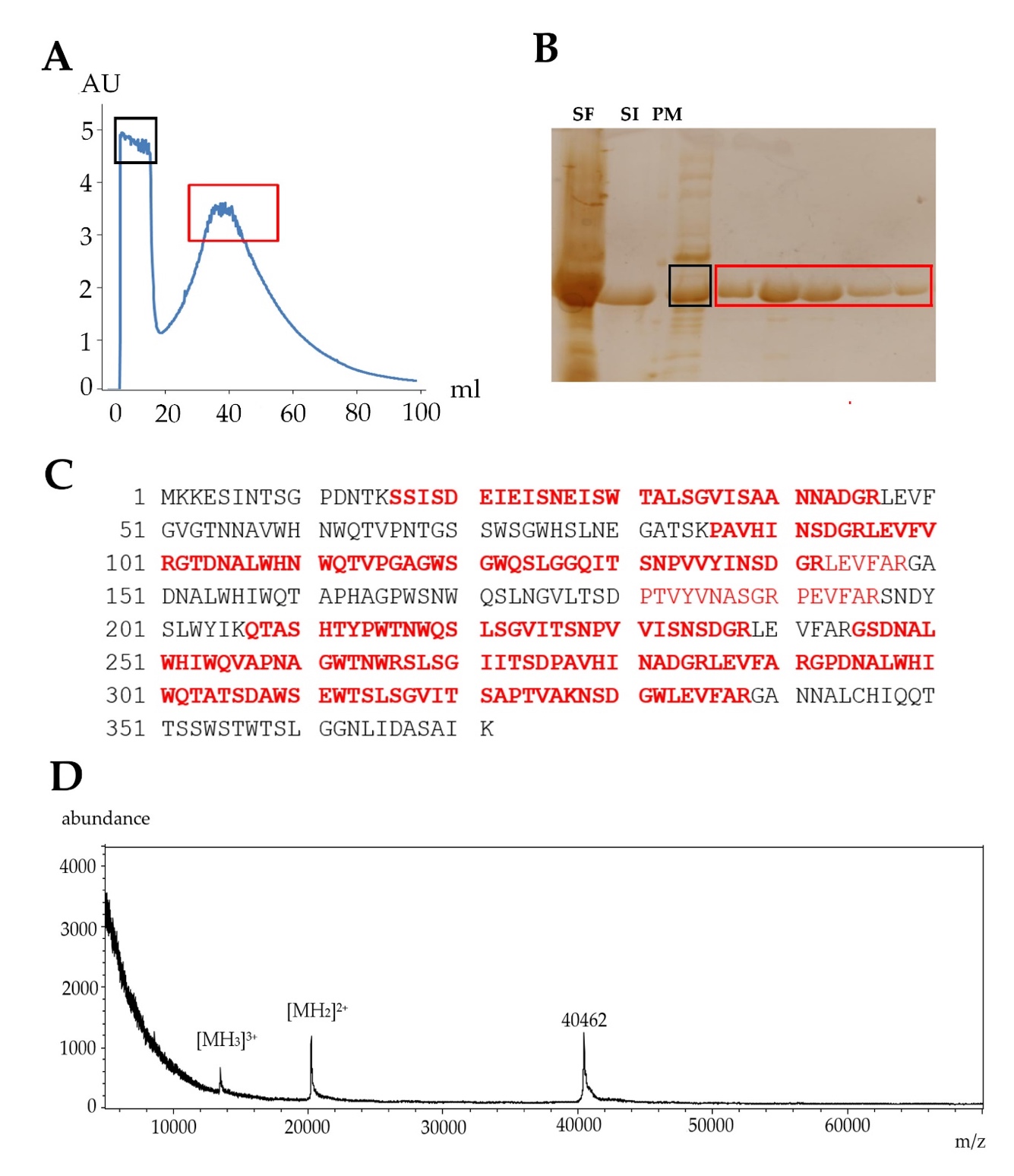


**Figure S1**. **Protein quality and identity**. (A) Representative record of PLL3 purification and (B) corresponding SDS-PAGE gel. SF – soluble fraction, SI – insoluble fraction PM – protein marker III (AppliChem). Fractions with PLL3 used for further analyses are depicted in the red rectangle. Polluted fractions are highlighted in the black rectangle. (C) Sequence coverage of PLL3 from MALDI MS/MS analyses. The confirmed section is in bold and red, uncertain sections are red, undetected sections are black. (D) Intact mass analyses of PLL3 revealed a major peak at 40,462 kDa, which corresponds to the full-length sequence including the initial methionine.

**
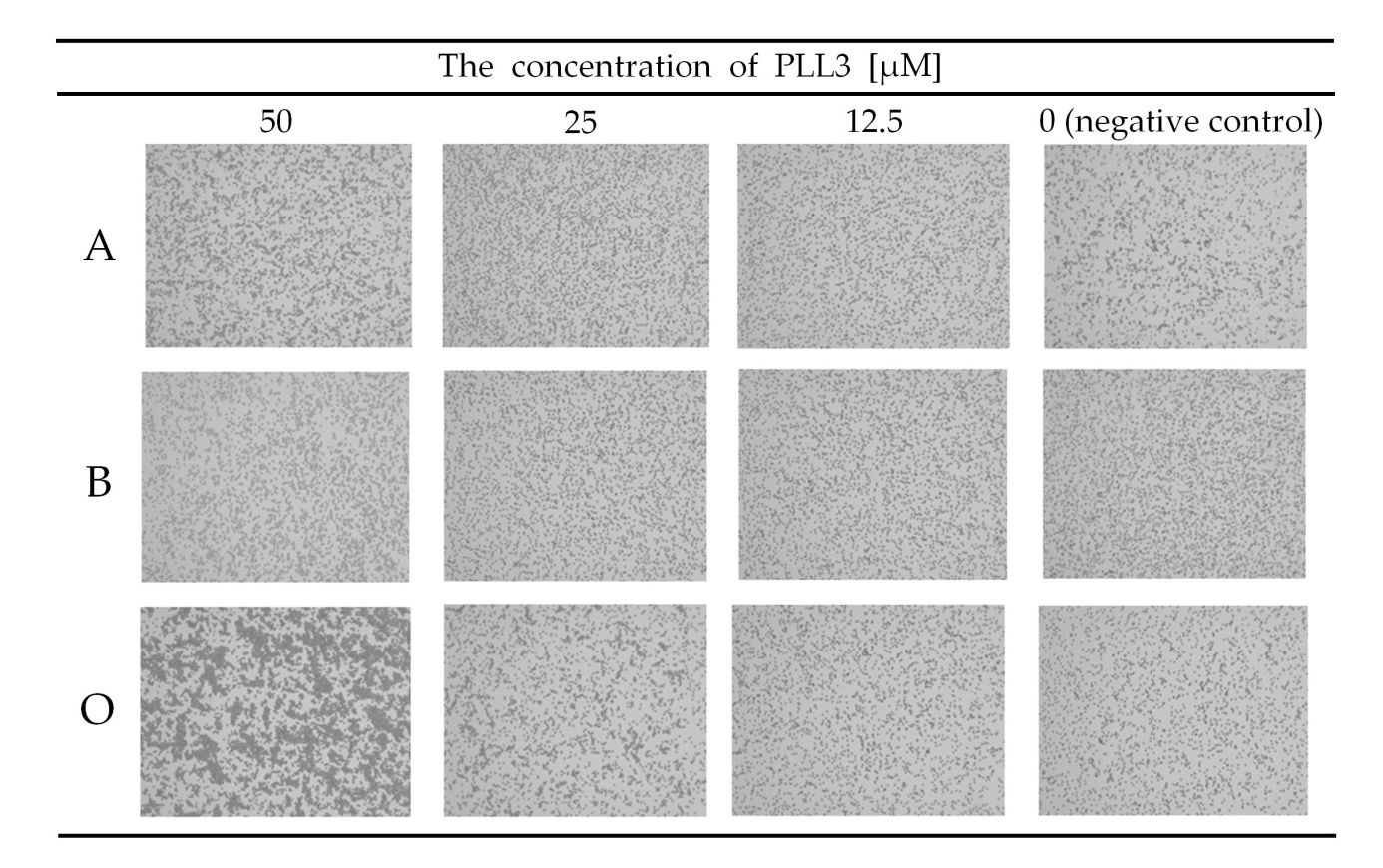
**

**Figure S2. The hemagglutination activity of PLL3 with erythrocytes of blood group A, B and O.** The erythrocytes were used as 10% (v/v). The PBS was used instead of protein solution in the last column (negative control). Only the erythrocytes of blood group O were agglutinated at the highest PLL3 concentration tested (50 μM). The experiment was observed at 200x magnification by optical microscope Olympus IX81.


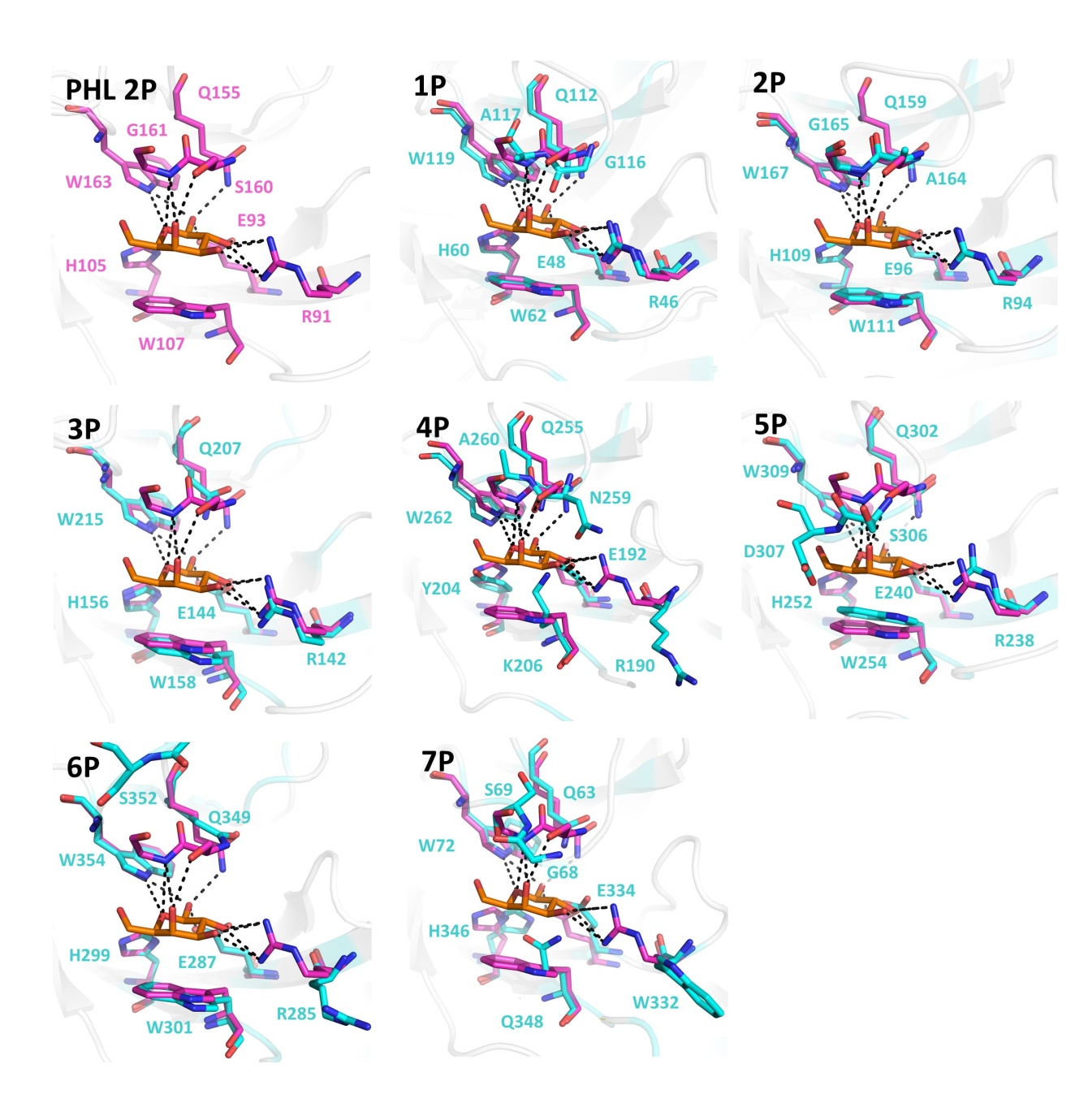


**Figure S3. Structural alignment of PLL3 putative polar binding sites with PHL polar binding site 2P**. PHL site 2P (PDB: 5MXH, previously designed 2G [15]) shown in magenta, putative polar binding site 1P – 7P of PLL3 shown in cyan and d-galactose from PHL complex (PDB: 5MXH) shown in orange. Polar contacts between d-galactose and PHL residues are represented by black dashed lines. Figures were generated in PyMOL (1.7.0.1).


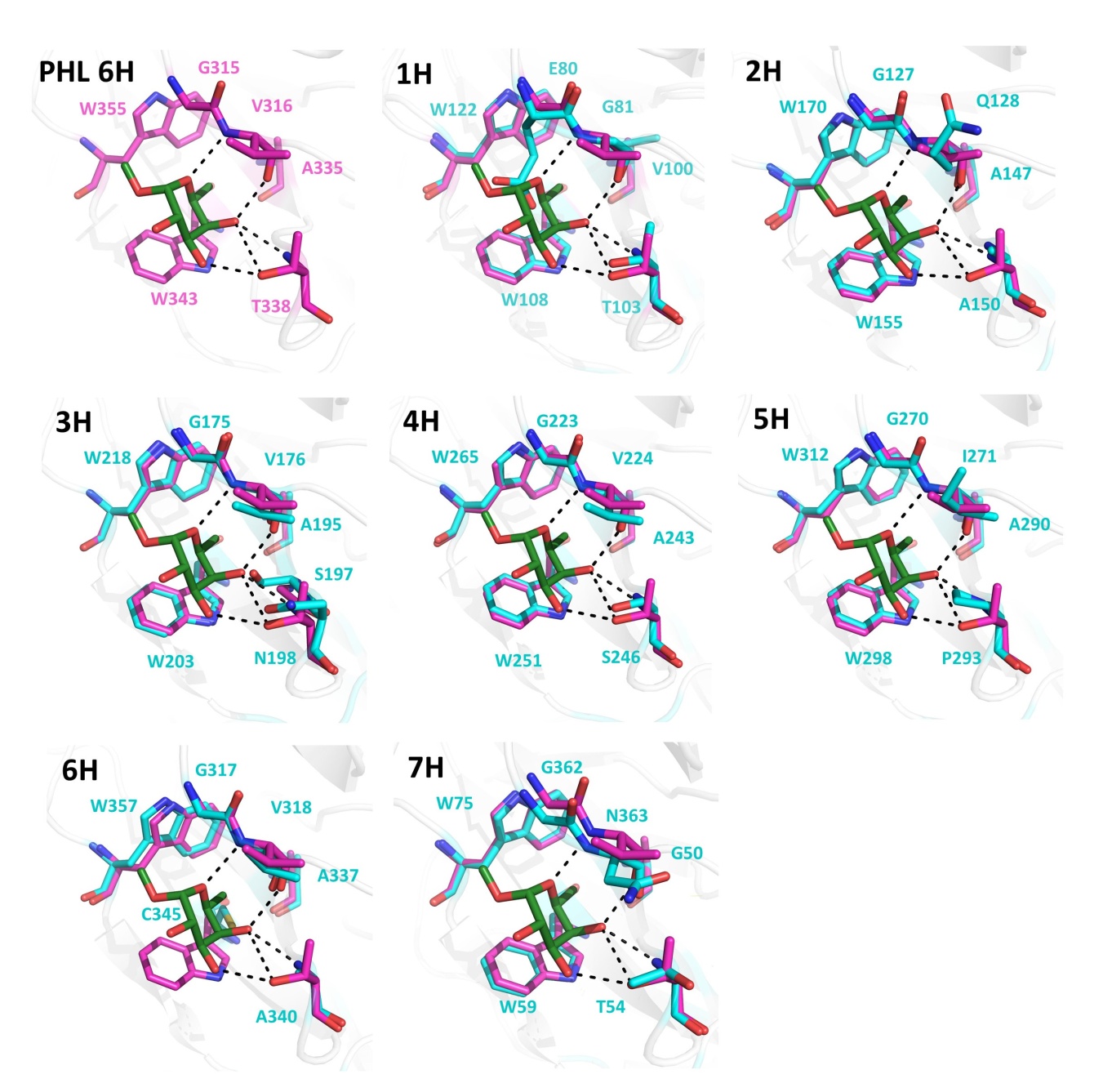


**Figure S4. Structural alignment of PLL3 putative hydrophobic binding sites with PHL polar binding site 6H.** PHL site 6H (PDB: 5MXH, previously designed 6F [15]) shown in magenta, putative polar binding site 1P – 7P of PLL3 shown in cyan and αMeFuc from PHL complex (PDB: 5MXH) shown in green. Polar contacts between αMeFuc and PHL residues are represented by black dashed lines. Figures were generated in PyMOL (1.7.0.1).
